# Improved production of class I lanthipeptides in *Escherichia coli*†

**DOI:** 10.1039/d2sc06597e

**Published:** 2023-02-13

**Authors:** Hyunji Lee, Chunyu Wu, Emily K. Desormeaux, Raymond Sarksian, Wilfred A. van der Donk

**Affiliations:** a Carl R. Woese Institute for Genomic Biology, University of Illinois at Urbana-Champaign 1206 W Gregory Drive Urbana Illinois 61801 USA vddonk@illinois.edu; b Department of Biochemistry, University of Illinois at Urbana–Champaign 600 South Mathews Avenue Urbana Illinois 61801 USA; c Department of Chemistry, The Howard Hughes Medical Institute, University of Illinois at Urbana–Champaign 600 South Mathews Avenue Urbana Illinois 61801 USA; d College of Pharmacy, Kyungsung University Busan 48434 Republic of Korea

## Abstract

Lanthipeptides are ribosomally synthesised and post-translationally modified peptides containing lanthionine (Lan) and methyllanthionine (MeLan) residues that are formed by dehydration of Ser/Thr residues followed by conjugate addition of Cys to the resulting dehydroamino acids. Class I lanthipeptide dehydratases utilize glutamyl-tRNA^Glu^ as a co-substrate to glutamylate Ser/Thr followed by glutamate elimination. Here we report a new system to heterologously express class I lanthipeptides in *Escherichia coli* through co-expression of the producing organism's glutamyl-tRNA synthetase (GluRS) and tRNA^Glu^ pair in the vector pEVOL. In contrast to the results in the absence of the pEVOL system, we observed the production of fully-dehydrated peptides, including epilancin 15X, and peptides from the *Bacteroidota Chryseobacterium* and *Runella*. A second common obstacle to production of lanthipeptides in *E. coli* is the formation of glutathione adducts. LanC-like (LanCL) enzymes were previously reported to add glutathione to dehydroamino-acid-containing proteins in Eukarya. Herein, we demonstrate that the LanCL enzymes can remove GSH adducts from *C*-glutathionylated peptides with dl- or ll-lanthionine stereochemistry. These two advances will aid synthetic biology-driven genome mining efforts to discover new lanthipeptides.

## Abstract 2

Expression of Glu-tRNA and its synthetase from lanthipeptide encoding bacteria using pEVOL improves production in *E. coli*. Often-observed glutathionylation can be reversed using LanCL enzymes.

## Graphical abstract

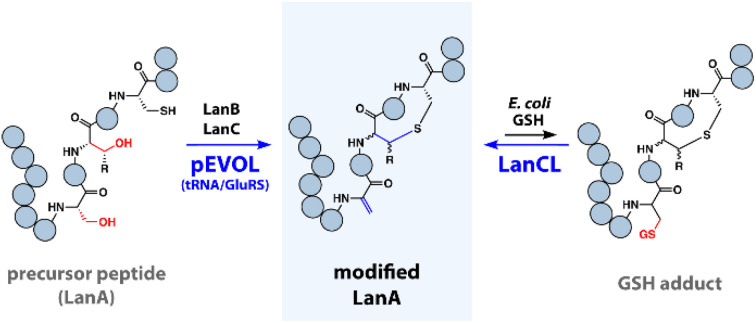

## Introduction

Ribosomally synthesised and post-translationally modified peptides (RiPPs) encompass an expanding class of natural products that have a wide range of biological functions, including antimicrobial, antifungal, antiviral, and virulence activity.^1,2^ RiPP biosynthesis features the translation of a genetically encoded precursor peptide, which comprises a C-terminal core peptide fused to an N-terminal leader peptide.^1,3^ The leader peptide is recognised by an array of modification enzymes, typically encoded in the same biosynthetic gene cluster (BGC), that install post-translational modifications (PTMs) on the core peptide. Following PTMs, the leader peptide is removed by a protease to reveal the final bioactive product. Because of the increasing availability of genomic databases and synthetic biology methodologies, the RiPP family is rapidly expanding, and several new members with unique structural properties and/or potent bioactivities have been recently unveiled.^3–14^

**Fig. 1:**
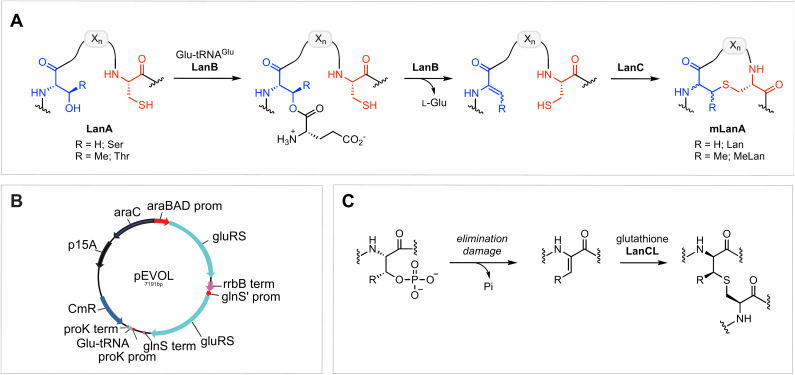
(A) Schematic representation of (methyl)lanthionine formation during the biosynthesis of class I lanthipeptides; different biosynthetic pathways result in different stereochemistry of the Dhb/Lan/MeLan. (B) pEVOL plasmid map with insertion of genes for GluRS and tRNA^Glu^. (C) LanCL-catalysed *C*-glutathionylation of a dehydroamino acid. R = H or Me.

Amongst the most well-studied classes of RiPPs are the lanthipeptides, which are characterised by intramolecular thioether linkages that are referred to as lanthionine (Lan) or methyllanthionine (MeLan) (Fig. 1A).^15^ The (Me)Lan structures are installed by one of five classes of lanthipeptide synthases (classes I–V), which dehydrate Ser and Thr residues in the LanA substrate peptides to generate dehydroalanine (Dha) and dehydrobutyrine (Dhb), respectively.^3,15–18^ Subsequently, Cys residues in the peptide react with Dha or Dhb *via* intramolecular Michael-type addition reactions to form Lan or MeLan resulting in the production of a polycyclic peptide termed mLanA (for modified LanA). Finally, a protease removes the leader peptide to produce the mature lanthipeptide. The five classes of lanthipeptides differ in the mechanisms of dehydration and cyclization.^3,15,19^

Class I lanthipeptide biosynthesis features a dehydratase LanB that activates and eliminates the side-chain hydroxyl groups of Ser and Thr residues, and a cyclase LanC that forms Lan or MeLan (Fig. 1A).^20,21^ LanB dehydratases utilize glutamyl-tRNA^Glu^ generated by GluRS to activate the Ser/Thr residues by transesterification, followed by glutamate elimination.^22,23^

While the potent antimicrobial and low resistance properties of class I lanthipeptides such as nisin have been exploited in the food industry for decades,^24^ the current need for new antibiotics has catalysed a flurry of recent studies on this class of peptides.^25^ To date, almost all class I lanthipeptides isolated were derived from the Gram-positive phyla *Bacillota* (formerly *Firmicutes*)^26^ and *Actinomycetota* (formerly *Actinobacteria*).^27,28^ Recent bioinformatic studies revealed that Gram-negative bacteria also encode a wealth of unexplored class I lanthipeptides in their genomes,^27,29^ which highlights the importance of investigations to discover novel class I lanthipeptides from more diverse organisms. The recent development of an automated and high throughput pipeline for RiPP discovery enables the identification of the products of BGCs *via* the heterologous expression of refactored BGCs in *E. coli*.^30^ While the expression of class II lanthipeptide BGCs exhibited the highest success rate of all RiPPs investigated and resulted in the discovery of new compounds active against ESKAPE pathogens, the expression of class I lanthipeptide BGCs encountered two major challenges that hampered further structural and biological studies.^30^

Although heterologous expression of a number of class I lanthipeptides has been successful in *E. coli*,^31–37^ many class I dehydratases fail to fully modify their substrates in this heterologous host. Previous studies have demonstrated that including GluRS and tRNA^Glu^ from the native lanthipeptide producing organism in the heterologous production system improves the production of the fully modified peptide.^23,34^ The observed improvement is likely due to the high degree of divergence in tRNA sequences of different phyla and the likelihood that LanB enzymes have coevolved to recognize specific tRNAs in the species of origin.^23^ Herein, we describe a new heterologous expression platform using the pEVOL vector^38^ to produce class I lanthipeptides in *E. coli* from both Gram-positive and Gram-negative origins. The engineered pEVOL vector encodes GluRS and tRNA^Glu^ from the native producer under both constitutive and inducible promoters.^38^ Three lanthipeptides, including epilancin 15X and peptides from *Chryseobacterium* and *Runella*, that failed to be fully dehydrated in previous attempts^30,33,39^ were successfully produced in *E. coli* using the new platform.

One of the other challenges during heterologous expression of class I lanthipeptides in *E. coli* is the formation of glutathione (GSH) adducts.^30,34,35^ Glutathione is a thiol-containing tripeptide that is involved in a variety of physiological functions in both mammals and bacteria.^40,41^ Due to the relatively high concentration of GSH in *E. coli*, which can accumulate to concentrations exceeding 10 mM,^42,43^ the thiol of GSH sometimes reacts with electrophilic dehydroamino acids in lanthipeptides. Whether this process is enzyme catalysed is not known. The addition of GSH to dehydroalanine residues has been previously observed in aged human lenses, and this type of non-reducible *C*-glutathionylation is often regarded as an “irreversible” process.^44–46^ GSH addition to dehydroamino-acid-containing proteins in mammals is mediated by LanC-like enzymes (LanCLs) with broad substrate scope (Fig. 1C).^47^ This activity is believed to remove damaged, deregulated proteins from the proteome. Since the reversibility of bacterial LanC was previously demonstrated,^48^ we envisioned that undesired glutathione molecules might be removed from peptides in synthetic biology studies by utilizing similar anticipated reversibility of LanCLs. In this study, we investigate the applicability of LanCLs to address the glutathionylation problem and show that GSH adducts can indeed be removed. The stereochemical requirements of deglutathionylation by LanCL2 was also examined.

## Results and discussion

### Heterologous expression of epilancin 15X derived from *Staphylococcus epidermidis* 15X154 using pEVOL

**Fig. 2:**
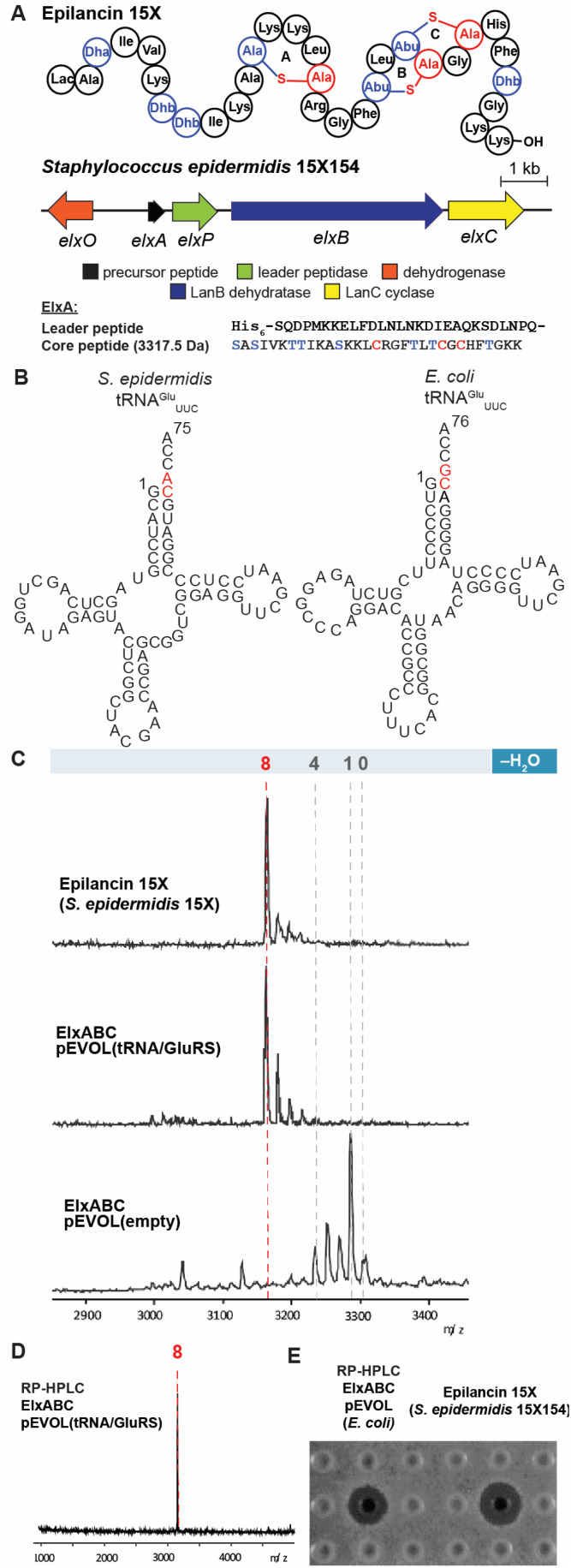
Heterologous expression of epilancin 15X using the pEVOL vector in *E. coli.* (A) Structure and biosynthetic gene cluster for epilancin 15X from *S. epidermidis* 15X154; Lac, lactyl; Abu, 2-aminobutyric acid. The same color coding is used as in Fig. 1A (B) Predicted cloverleaf structures of *S. epidermidis* 15X154 and *E. coli* tRNA^Glu^. (C) MALDI-TOF MS analysis of epilancin 15X isolated from *S. epidermidis* 15X154 (top), and His_6_-ElxA isolated from the co-expression with pRSF.elxBC with (middle) or without (bottom) using the pEVOL platform in *E. coli* after *in vitro* leader peptide removal by ElxP. Observed and calculated masses for each compound are listed in Table S1.† (D) MALDI-TOF MS analysis of HPLC-purified fully dehydrated ElxA after *in vitro* leader peptide removal by ElxP. Calculated *m*/*z* 3172, Observed 3172; (E) zone of growth inhibition of purified epilancin 15X from panel (D) and epilancin 15X isolated from *S. epidermidis* 15X154 against *S. carnosus* TM300.

The class I lanthipeptide epilancin 15X (Fig. 2A) was isolated from *Staphylococcus epidermidis* 15X154 and is active against a subset of Gram-positive bacteria, including methicillin-resistant *S. aureus* (MRSA) and vancomycin-resistant *Enterococci* (VRE), with MIC values among the lowest reported for lanthipeptides.^49^ These findings suggest the molecule might have a novel target,^2^ but SAR studies have been hampered by the inability of a robust heterologous expression platform (see below). The epilancin 15X gene cluster contains five genes involved in its biosynthesis (elxABCOP) (Fig. 2A).^39^ The precursor peptide ElxA is modified by the dehydratase ElxB, followed by the cyclase ElxC that forms Lan and MeLan crosslinks. Next, the protease ElxP removes the leader peptide, and the dehydrogenase ElxO converts the N-terminal Dha to a lactyl residue to form the mature epilancin 15X (Fig. 2A).^39^

The wild-type epilancin 15X isolated from the producing organism is eight-fold dehydrated compared to the precursor peptide ElxA (Fig. 2A). A number of studies have attempted to elicit heterologous production of epilancin 15X, but none were able to obtain the bioactive fully-dehydrated peptide as a major product. In 2012, Vélasquez *et al.* reported a mixture of one to five-fold dehydrated ElxA when co-expressed with ElxB and ElxC in *E. coli.*^39^ In 2019, Van Staden *et al.* reported co-expressing the epilancin 15X precursor peptide fused to a green fluorescent protein with the dehydratase and cyclase from the nisin operon.^33^ The resulting products consisted of a mixture of dehydration states with the fully dehydrated peptide a minor product and lacking antimicrobial activity.^33^ Given the previous difficulties in expressing functional epilancin 15X in *E. coli*, we pursued a different strategy (Fig. S1, ESI†). Our initial attempts to obtain fully modified His_6_-tagged ElxA through co-expression with ElxB and ElxC in *E. coli* were unsuccessful, resulting in four out of eight dehydrations of ElxA (Fig. S2, ESI†). The poor ElxB activity is likely the result of differences between the tRNA^Glu^ and GluRS sequences in *E. coli* and *S. epidermidis* 15X154; the latter is only 33% identical to *E. coli* GluRS. In addition, nucleotides in the tRNA^Glu^ acceptor stem highlighted in red, which are major recognition elements of the lantibiotic dehydratases,^23^ are different in *E. coli* and *S. epidermidis* 15X154 (Fig. 2B). These differences could explain why the dehydration catalysed by ElxB was not efficient. The number of dehydrations improved to five after incorporating both *S. epidermidis* tRNA^Glu^ and GluRS under control of the T7 promoter in the co-expression system (Fig. S2, ESI†).

To further improve the ElxB dehydratase activity, we introduced a derivative of the vector pEVOL that co-expresses GluRS and tRNA^Glu^ from *S. epidermidis* (*Se*) in the class I lanthipeptide heterologous expression system. The pEVOL vector was constructed previously for enhancing unnatural amino acid incorporation into proteins using aminoacyl-tRNA synthetase (aaRS)/suppressor tRNA pairs.^38^ In this study, a copy of the GluRS gene from the lanthipeptide-encoding producing organism was introduced under an inducible araBAD promotor in the pEVOL plasmid (Fig. 1B). A second copy of the GluRS gene was placed under the constitutive glnS′ promoter to further increase protein yields by creating a basal level of *Se*-GluRS at the start of induction (Fig. 1B). Additionally, the *Se*-tRNA^Glu^ sequence from the lanthipeptide-encoding producer organism was cloned such that the promoter proK controlled its expression constitutively (Fig. 1B). The sequence of the assembled plasmid was confirmed by DNA sequencing and the plasmid was used to transform the *E. coli* expression host.

The pEVOL vector was used in combination with plasmids expressing the lanthipeptide precursor peptide, dehydratase, and cyclase under control of T7 promotors in *E. coli* BL21 (DE3) cells (Fig. S1, ESI†). The transformed cells were incubated at 37 °C with shaking and treated with l-arabinose at the beginning of the exponential growth phase to induce production of GluRS. The culture was then placed in a 20 °C incubator for slow growth and shaken for at least 2 h until the culture reached the end of the exponential growth phase. The second inducer isopropyl β-d-1-thiogalactopyranoside (IPTG) was added to the culture to induce expression of the epilancin 15X biosynthetic machinery, and the culture was incubated with shaking for an additional 15 h. During the expression procedure, allowing at least 2 h of “slow growing time” between the addition of the two inducers (l-arabinose and IPTG) was critical for the yield of the fully modified product. After the co-expression, the modified His_6_-tagged precursor peptide was isolated from cells and purified by immobilised metal ion affinity chromatography (IMAC). The post-translationally modified peptide was further purified using reversed-phase high-performance liquid chromatography (RP-HPLC) and analysed by matrix-assisted laser desorption/ionization time-of-flight mass spectrometry (MALDI-TOF MS).

After *in vitro* leader peptide removal with ElxP protease,^50^ a mixture of five to eight-fold dehydrated peptides was observed with the desired eight-fold dehydrated peptide as the major product (Fig. 2C). We were able to isolate the fully dehydrated peptide by RP-HPLC (Fig. 2D), and the purified peptide had similar bioactivity and ESI-HRMS spectrum as the wild-type epilancin 15X isolated from the producing organism (Fig. 2E and S3, ESI†). The successful production of bioactive epilancin 15X using the pEVOL platform implies that the timing and quantity of tRNA^Glu^ and GluRS are important for successful production.

### Heterologous expression of new class I lanthipeptides derived from Gram-negative bacteria using pEVOL

**Fig. 3:**
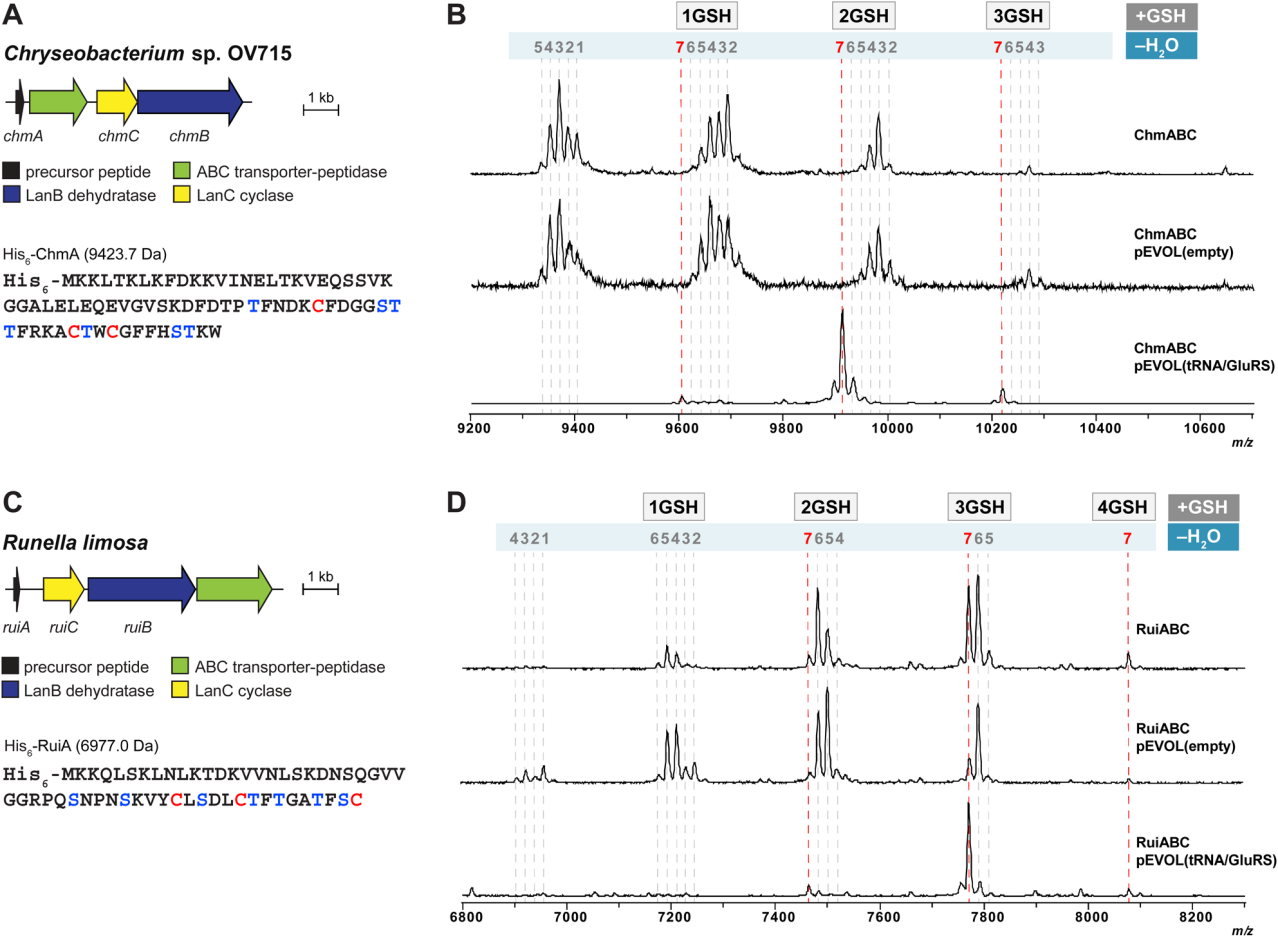
(A) Lanthipeptide biosynthetic gene cluster from *Chryseobacterium* sp. OV715 (WP_047454108 for ChrC). Ser/Thr and Cys residues in the putative core region of the precursor peptide are shown in blue and red, respectively. (B) MALDI-TOF MS data from heterologous expression of the refactored gene cluster from *Chryseobacterium* sp. OV715 using the pEVOL platform in *E. coli*. pEVOL(tRNA/GluRS) contained tRNA^Glu^ and GluRS genes from *Chryseobacterium* sp. OV715. Observed and calculated masses for each compound are listed in Table S2.† (C) Biosynthetic gene cluster from *Runella limosa* (WP_157607478). Ser/Thr and Cys residues in the putative core region of the precursor peptide are shown in blue and red, respectively. (D) MALDI-TOF MS data from heterologous expression of the refactored lanthipeptide biosynthetic gene cluster from *R. limosa* using the pEVOL platform in *E. coli*. pEVOL(tRNA/GluRS) contained tRNA^Glu^ and GluRS genes from *Chryseobacterium* sp. OV715. Observed and calculated masses for each compound are listed in Table S3.†

After demonstrating the utility of the pEVOL tRNA/GluRS system with the epilancin 15X biosynthetic machinery encoded by the Gram-positive strain *S. epidermidis* 15X, we switched our attention to two biosynthetic gene clusters encoded in the genomes of the Gram-negative bacteria *Chryseobacterium* OV715 and *Runella limosa* from the phylum *Bacteroidota* (formerly *Bacteriodetes*) (Fig. 3). These BGCs were previously identified through bioinformatic genome mining efforts and are predicted to encode new class I lanthipeptides.^27^ Attempts to obtain the products of these BGCs by using the automated refactoring system were not successful.^30^ Since nearly all known class I lanthipeptides are produced by Gram-positive bacteria, the discovery of novel class I lanthipeptides from Gram-negative bacteria would be valuable for investigating new structures and potentially new biological functions.

When we tested heterologous expression of each refactored gene cluster containing the hypothetical LanA, LanB, and LanC-encoding genes, a mixture of peptides was produced with partial modifications ranging between two to seven dehydrations (Fig. 3B and D). Furthermore, groups of the partially dehydrated peptides were observed by MALDI-TOF MS with multiple increases of 307 Da, which suggests the addition of glutathione (GSH) to the reactive dehydroamino acids of the dehydrated peptides.^30,34,35^ This result showed that, like the observations with epilancin 15X, the LanB dehydratases from these BGCs cannot effectively utilize the tRNA^Glu^/GluRS system from *E. coli* to activate Ser or Thr residues by glutamylation. Indeed, the tRNA^Glu^ sequence in *E. coli* at the critical recognition positions is divergent compared to those of *Chryseobacterium* and *Runella* (Fig. S4, ESI†). This observation led us to incorporate the tRNA^Glu^/GluRS pair from the producing organism during the expression process.

Heterologous expression of the *Chryseobacterium* ChmABC in *E. coli* was performed using the pEVOL system containing tRNA^Glu^/GluRS from the producer (Fig. 3B). When the empty pEVOL plasmid without native GluRS and tRNA^Glu^ was used, a mixture of partially dehydrated peptides was observed, similar to the result obtained without the pEVOL plasmid. On the other hand, when the native tRNA^Glu^/GluRS system was introduced, seven dehydrations of the precursor peptide were observed (Fig. 3B and S5, ESI†). It should be noted that the products were still obtained as glutathionylated peptides. *N*-ethylmaleimide (NEM) and dithiothreitol (DTT) assays were then performed to test for free Cys and dehydroamino acids in the peptide.^51^ The results suggested that all Cys residues in the peptide participated in ring formation (Fig. S6, ESI†). Of the seven dehydroamino acids, three reacted intramolecularly with Cys in the peptide and two intermolecularly with GSH in the cell; the remaining two dehydroamino acids reacted with DTT in the *in vitro* assay (Fig. S6, ESI†). Tandem MS experiments revealed the location of the GSH adducts and the likely ring pattern of the product (Fig. S7, ESI†).

Heterologous expression of RuiABC from *Runella limosa* was tested next using the pEVOL platform (Fig. 3D). Because of the sequence similarity between the tRNA^Glu^ from *Runella* and *Chryseobacterium* (Fig. S4, ESI†), we first tested whether the pEVOL plasmid encoding the tRNA^Glu^ and GluRS from *Chryseobacterium* was suitable. As anticipated, LanB from the *Runella* species accepted the glutamyl-tRNA from *Chryseobacterium,* which resulted in improved dehydration activity such that the 7-fold dehydrated peptide (mRuiA) was the major product (Fig. 3D and S8, ESI†). NEM and DTT assays of the peptides and tandem MS analysis confirmed the presence of three thioether rings, and four dehydroamino acids, three of which had reacted with GSH molecules in the cell resulting in the DTT assay providing only one additional adduct (Fig. 3D, S9 and S10, ESI†). Overall, these results clearly showed that the dehydration reaction is facilitated by the addition of the tRNA^Glu^/GluRS pair in the pEVOL platform during the heterologous expression of class I lanthipeptides from various phyla in *E. coli*. However, the observed glutathionylation provided an obstacle for obtaining the native products.

### Deglutathionylation utilizing LanCL enzymes

Glutathionylation of reactive dehydroamino acids in lanthipeptides is often observed during heterologous expression in *E. coli*,^30,34,35^ impeding further structural and biological investigations. Efforts to optimize expression conditions to prevent glutathionylation, such as using different *E. coli* strains or changing the expression temperature, failed to prevent glutathionylation. Developing a novel strategy that can remove undesirable GSH from glutathionylated lanthipeptides would therefore be valuable.

**Fig. 4:**
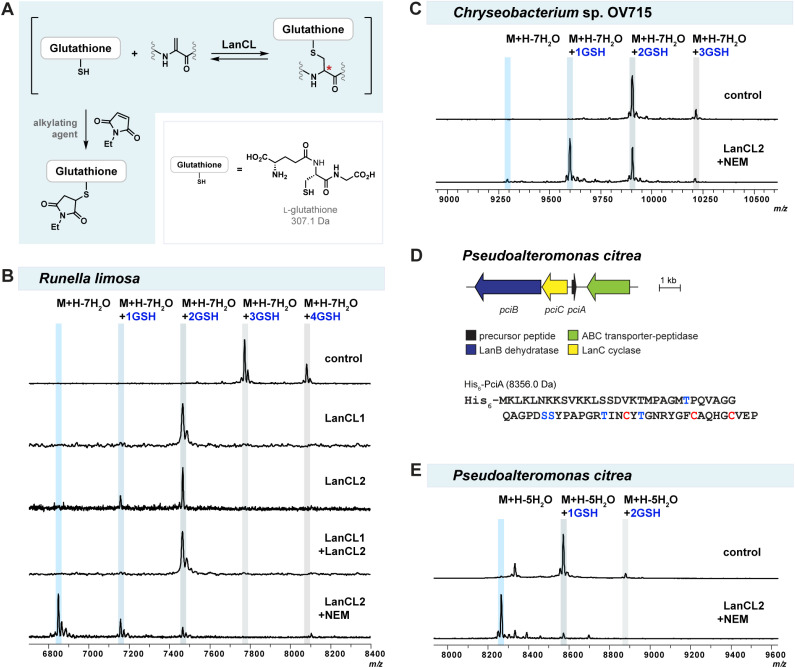
(A) Reversible glutathionylation/deglutathionylation catalyzed by LanCLs. An alkylating agent such as *N*-ethylmaleimide (NEM) was used to trap the free thiol of GSH. (B) MALDI-TOF MS data of LanCL-mediated deglutathionylation of modified RuiA; (C) MALDI-TOF MS data of LanCL-mediated deglutathionylation of modified ChmA. (D) Biosynthetic gene cluster from *Pseudoalteromonas citrea* DSM 8771 (WP_010365947.1 for PciC). Ser/Thr and Cys residues participating in post-translational modifications are shown in blue and red, respectively. (E) MALDI-TOF MS data of LanCL-mediated deglutathionylation of modified PciA.

Mammalian LanCL proteins share structural similarities with bacterial LanC cyclases.^52,53^ Both classes of enzymes mediate C–S bond formation through conjugate addition of a thiol group to a dehydroamino acid, but LanCLs use the thiol of GSH for intermolecular reaction rather than Cys residues for intramolecular cyclization (Fig. 1A and C). LanCLs exhibit considerable substrate tolerance and were shown to add GSH to Dha-containing proteins MEK1 and ERK1 as well as prochlorosin and cytolysin derived peptides.^47^ In addition, a previous study demonstrated that bacterial LanC enzymes can reverse the cyclization by deprotonating at the α-position of (methyl)lanthionine residues in peptide products and opening the ring structures *via* a retro-Michael process.^48^ Based on these findings, we hypothesised that LanCLs might also be able to reverse the thia-Michael addition and remove GSH from glutathionylated adducts (Fig. 4A).

To test the reversibility and substrate tolerance of LanCLs, we first reacted glutathione-conjugated mRuiA with LanCLs *in vitro* and analysed the degree of elimination of GSH by MALDI-TOF MS. Glutathione removal was observed in reactions with either human LanCL1 or LanCL2, indicating LanCLs accepted the glutathionylated mRuiA as a substrate and catalysed the retro Michael-type reaction (Fig. 4B). When both LanCL1 and LanCL2 were co-incubated with the substrate, no synergistic effect in GSH elimination was observed (Fig. 4B). A dithiothreitol (DTT) assay was performed to see whether the LanCL-mediated deglutathionylation of *Runella* peptides indeed produced dehydroamino acids. After the treatment with LanCL1, DTT was added to the reaction mixture and analysis by MALDI-TOF MS clearly demonstrated generation of the dehydroamino acids that reacted with DTT (Fig. S12, ESI†).

We noted that two out of the four GSH molecules conjugated to the peptide were readily removed with LanCL enzymes, but that two GSH adducts remained (Fig. 4B). Since the addition and elimination of glutathione is likely an equilibrium process, we attempted to force the equilibrium toward the removal of GSH by capturing the free thiol of GSH with an alkylating agent such as NEM, *S*-methyl methanethiosulfonate (MMTS), or iodoacetic acid (IAA) (Fig. 4A). The addition of NEM provided the best result and indeed facilitated the elimination reaction to produce non-glutathionylated mRuiA (Fig. 4B). To further simplify the deglutathionylation experimental procedure, we co-expressed all three plasmids together in *E. coli*: pET28(RuiABC), tRNA^Glu^/GluRS-containing pEVOL, and pET28(LanCL2) (Fig. S13, ESI†). The His-tagged mRuiA and LanCL2 were co-purified by metal affinity chromatography. When the obtained mRuiA/LanCL2 mixture was diluted with Tris buffer and incubated with NEM, all four glutathione molecules were removed from the modified RuiA (Fig. 4B). This result showed that LanCL2 can be co-expressed with LanABC and the pEVOL platform to eliminate GSH from mLanA, which facilitates production of desired class I lanthipeptides.

Having observed the successful deglutathionylation ability of LanCLs, we also tested removal of GSH adducts produced by heterologous expression of lanthipeptides from *Chryseobacterium* OV715 and *Pseudoalteromonas citrea* DSM8771 (*pci*) in *E. coli* (Fig. 4C and E). The pEVOL plasmid was not needed for modification of PciA because the tRNA^Glu^ sequence in *P. citrea* is similar to that of *E. coli* (Fig. S4†). Similar to the results with mRuiA, the elimination of GSH from the mChmA and mPciA adducts was also observed. However, the efficiency of the deglutathionylation for these peptides was variable, with near full removal for the lanthipeptide from *P. alteromonas* but incomplete removal for the peptide from *Chryseobacterium*. These results suggest that the deglutathionylation might depend on the position of the GSH molecules on the peptides and/or the stereochemistry of the adducts.

To gain molecular insight into the mechanism of GSH removal by LanCLs, we tested deglutathionylation activity by a LanCL2 mutant. A histidine residue in NisC at position 212, which is conserved in LanCLs (His219 in LanCL1; His264 in LanCL2), has been proposed to be involved in protonation of the enolate intermediate during the intramolecular cyclization event.^54^ Site-directed mutagenesis on the corresponding His residues in LanCLs abolished GSH addition activity, suggesting that they might also be crucial for the enolate protonation during intermolecular C–S bond formation (Fig. 1C).^47^ To investigate whether His264 in LanCL2 is important for C–S bond breakage in the retro-thia-Michael reaction, *in vitro* deglutathionylation using mRuiA as a substrate with LanCL2-H264A was investigated. The MALDI-TOF mass spectrum of the reaction showed that LanCL2-H264A was unable to remove GSH from mRuiA compared to the wild-type LanCL2 (Fig. S14, ESI†). This result indicates that the conserved His plays an important role during deglutathionylation, where it might act as a base to abstract the proton from the α-carbon to facilitate the retro-thia-Michael reaction.

### Stereochemistry of LanCL2-mediated deglutathionylation

**Fig. 5:**
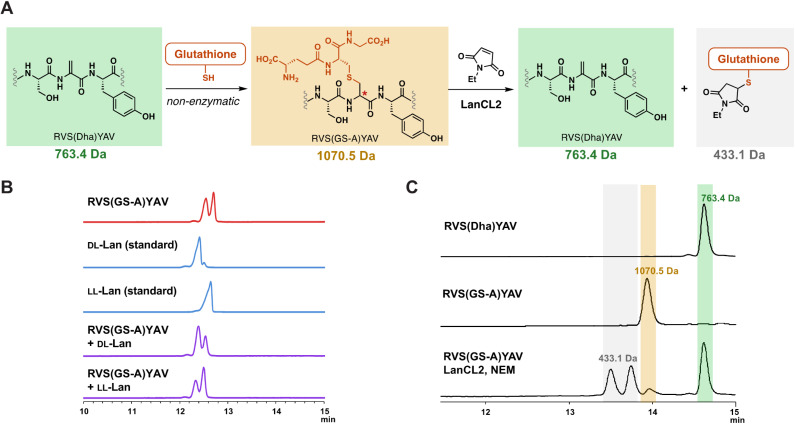
(A) Schematic representation of non-enzymatic glutathionylation of RVS(Dha)YAV 7-mer peptide followed by LanCL2-catalysed deglutathionylation. Calculated [M + H]^+^ mass for each structure is noted. (B) Chiral GC-MS analysis of RVS(GS-A)YAV after hydrolysis and derivatisation (red). Derivatised Lan was detected at *m*/*z* 365; dl-Lan and ll-Lan standards after derivatisation are shown in blue. Hydrolysed and derivatised RVS(GS-A)YAV sample was co-injected with each standard (purple). (C) LC-MS analysis of RVS(Dha)YAV, RVS(GS-A)YAV, and the LanCL2-treated reaction mixture. Selective-ion monitoring (SIM) with electrospray ionization was used to detect RVS(Dha)YAV (*m*/*z* = 763.4), RVS(GS-A)YAV (*m*/*z* = 1070.5), and glutathionylated NEM (*m*/*z* = 433.1).

With the observation that LanCLs can accept glutathionylated peptides as substrates, we next investigated the stereochemical preference of deglutathionylation by LanCL2. Previous research showed that LanCL2 added GSH to the Dhb of a synthetic peptide mimicking the activation loop of Erk in a stereoselective manner, generating a d-stereocenter at the α-position of the former Dhb.^47^ To test whether the reverse reaction by LanCL2 is also stereoselective, we prepared a glutathionylated peptide as a d/l mixture by non-enzymatic GSH addition and treated the peptide with LanCL2 (Fig. 5A). The Dha-containing heptamer peptide (RVS(Dha)YAV) was synthesised by solid phase peptide synthesis similar to a reported procedure,^55^ and GSH was added to the peptide at basic pH. This process generates a lanthionine linkage between the two peptides (Fig. 5A). To confirm the generation of an epimeric mixture of glutathionylated peptides, gas chromatography-mass spectrometry (GC-MS) analysis was performed with a chiral stationary phase.^56^ After the glutathionylation, the resulting peptide was hydrolysed, and the amino acids derivatised and analysed (Fig. 5B).^57–59^ The GC-MS trace showed two peaks of derivatised lanthionine indicating the non-enzymatic glutathionylation indeed yielded two diastereomers. Co-injection of the peptide with ll-Lan or dl-Lan synthetic standards confirmed the presence of two diastereomers.

Next, the mixture of epimeric glutathionylated heptamer peptides was treated with LanCL2 and NEM to investigate the stereochemical preference of deglutathionylation. LanCL2 accepted the glutathionylated synthetic peptides as substrate, and the removal of GSH was observed by MALDI-TOF MS (Fig. S15, ESI†). To quantitatively track the conversion from the glutathionylated peptide to the Dha-containing peptide, the peptide samples and the LanCL2 reaction mixture were analysed by liquid chromatography-mass spectrometry (LC-MS) with selected ion monitoring (SIM) for three ions, the glutathionylated peptide, the deglutathionylated product, and the alkylated GSH (Fig. 5A and C). After the treatment of RVS(GS-A)YAV (GS-A = glutathione adduct to Dha) with LanCL2 and NEM for two hours, nearly all of the glutathionylated peptide (1070.5 Da) disappeared and the Dha-containing heptamer (763.4 Da) was generated. At the same time, the production of the glutathionylated NEM adduct (433.1 Da) was observed in the spectrum as two diastereomers resulting from a new stereogenic center on the NEM moiety. This result indicates that LanCL2 can accept both glutathionylated peptides with a dl- or ll-lanthionine stereochemistry, which is different from the observation where dl-MeLan formation is preferred over ll-MeLan formation by LanCL2 during glutathionylation. Although unanticipated, from the viewpoint of utility of LanCL enzymes, this observation is encouraging since addition of GSH to lanthipeptides in *E. coli* may be non-stereoselective. In turn, these findings suggest that the inability to fully remove GSH from the lanthipeptide from *Chryseobacterium* (Fig. 4C) is likely the result of inaccessibility of some of the glutathionylation sites.

## Conclusion

A growing number of new lanthipeptides are being discovered and identified through genome mining, which offers exciting opportunities to explore novel structural features, biological functions, as well as bioengineering potential for modifying enzymes. To obtain products of lanthipeptide BGCs and investigate their physicochemical and biological properties, heterologous expression of lanthipeptide BGCs in a genetically tractable host is highly beneficial. BGCs of class I lanthipeptides contain LanBs that dehydrate Ser and Thr in a tRNA-dependent manner to yield Dha or Dhb motifs. The post-translational modification process often does not proceed smoothly in a heterologous expression setting, with two major issues having been identified: (1) incomplete dehydration due to the incompatibility of *E. coli* tRNA^Glu^ for efficient LanB catalysis, and (2) intermolecular GSH addition to electrophilic Dha/Dhb of a modified lanthipeptide. Here, we developed a pEVOL-derived platform that generates glutamyl-tRNA from the original producing organism prior to expression of the biosynthetic enzymes. We demonstrate that this platform improves dehydration efficiency for systems that previously resulted in incomplete dehydration in *E. coli.* We also showed the utility of mammalian LanCL enzymes to remove unwanted GSH from modified LanAs. These strategies will facilitate genome mining for class I lanthipeptides by heterologous expression in *E. coli* and preparation of analogues for SAR studies.

## Data availability

Primary data associated with this study can be found at: Mendeley Data, V1, DOI: 10.17632/cnb94ggsf8.1

## Author contributions

C. W. and H. L. contributed equally to this work. Credit: Chunyu Wu investigation; methodology; visualization; writing-original draft, Hyunji Lee investigation; methodology; visualization; writing-original draft, Emily K. Desormeaux investigation, Raymond Sarksian investigation, and Wilfred A. van der Donk supervision, conceptualization, writing-original draft.

## Conflicts of interest

There are no conflicts to declare.

## Supplementary Material

SC-014-D2SC06597E-s001
