# Supplementary material for: Improved production of class I lanthipeptides in Escherichia coli
Source: Chem Sci. 2023 Feb 13;14(10):2537–46. doi: 10.1039/d2sc06597e (PMC9993889; doi:10.1039/d2sc06597e)
Supplement: SC-014-D2SC06597E-s001 [file SC-014-D2SC06597E-s001.pdf]

Electronic Supplementary Material (ESI) for Chemical Science.
This journal is © The Royal Society of Chemistry 2023

Electronic Supplementary Information

Improved Production of Class I Lanthipeptides in *Escherichia coli*

Hyunji Lee,[a,d,§] Chunyu Wu,[b,§] Emily K. Desormeaux,[c] Raymond Sarksian,[c] and Wilfred A. van der Donk[a-c,*]

[a] *Carl R. Woese Institute for Genomic Biology, University of Illinois at Urbana-Champaign, 1206 W Gregory Drive, Urbana, Illinois 61801, United States*
[b] *Department of Biochemistry, University of Illinois at Urbana–Champaign, 600 South Mathews Avenue, Urbana, Illinois 61801, United States.*
[c] *Department of Chemistry, and the Howard Hughes Medical Institute, University of Illinois at Urbana–Champaign, 600 South Mathews Avenue, Urbana, Illinois 61801, United States.*
[d] *College of Pharmacy, Kyungsung University, Busan 48434, Republic of Korea.*
[§] *These authors contributed equally to this study*

Primary data associated with this study can be found at:

Mendeley Data, V1, doi: 10.17632/cnb94ggsf8.1

Experimental procedures:

General methods

Chemical reagents and media components used in this study were purchased from Sigma-Aldrich or Thermo Fisher Scientific, unless otherwise specified. Oligonucleotides and enzymes were purchased from Integrated DNA Technologies (IDT) and New England Biolabs (NEB), respectively. Polymerase chain reaction (PCR) amplifications were carried out using Q5 polymerase (NEB) on an automated thermocycler (C1000, Bio-Rad). DNA sequencing was performed using appropriate primers by ACGT, Inc. MALDI-TOF MS analyses were conducted at the Mass Spectrometry Facility at UIUC using a Bruker UltrafleXtreme MALDI TOF/TOF spectrometer (Bruker Daltonics). For MALDI−TOF MS analysis, samples were desalted using ZipTipC18 (Millipore) and spotted onto a MALDI target plate with a matrix solution usually consisting of a saturated aqueous solution of super DHB (2,5-dihydroxy benzoic acid; Sigma-Aldrich). Peptide purification was performed by reversed-phase high performance liquid chromatography (RP−HPLC) on an Agilent 1260 Infinity II instrument equipped with a Macherey-Nagel C18 reversed-phase column (4.6 mm i.d. × 250 mm L). For RP−HPLC, solvent A was 0.1% TFA in H_2O, and solvent B was acetonitrile containing 0.1% trifluoroacetic acid (TFA). An elution gradient from 0% solvent B to 100% solvent B over 30 min was used unless specified otherwise.

Plasmid construction for heterologous expression of epilancin 15X

The plasmids constructed for this study are listed in Table S4 and their combined application for peptide production and purification is shown in Figure S1. The $tRNA^{Glu}$ sequences were identified using the algorithm tRNAscan-SE.[1, 2] The genes *elxB, elxC, elxP*, and *S. epidermidis* GluRS were amplified from genomic DNA purified from *S. epidermidis* 15X154 with the PureLink^{TM} genomic DNA mini kit (Invitrogen), according to the manufacturer's recommendations, and using primers possessing the required restriction sites (Table S5). The genes encoding His_6-ElxA and *S. epidermidis* $tRNA^{Glu}$ were

constructed using synthetic gene fragment gBlocks (IDT), and amplified using primers for inserts and plasmid backbones (Table S5).

The recipient vectors (pET21a, pRSFDuet-1, and pEVOL) were purified with the Miniprep kit (Qiagen). PCR products and vectors were digested with the appropriate restriction enzymes (NEB) (Table S5) according to the manufacturer's recommendations and separated by agarose gel electrophoresis. The digested PCR products and vectors were excised and purified from the gels with the GeneJET gel extraction kit (Thermo Scientific). Gibson ligation was performed in a 10 µL reaction containing 50 ng of the vector and the insert at a molar ratio of vector:insert of 1:3 with HiFi Gibson Assembly Master Mix for 1 h at 50 °C. *E. coli* DH5α cells (NEB) were transformed with the Gibson mix and colonies were selected on Luria-Bertani (LB) agar plates supplemented with 50 µg/mL ampicillin (pET21a and pETDuet-1), 50 µg/mL kanamycin (pRSFDuet-1), and 30 µg/mL chloramphenicol (pEVOL). Colonies were selected for plasmid extraction, and the plasmids were purified with the Miniprep kit (Qiagen) and the plasmid sequence was validated by ACGT, Inc.

Heterologous expression of peptides with pEVOL plasmids
Chemically competent *E. coli* BL21 (DE3) cells were transformed with combinations of the plasmids prepared (Figure S1) to co-express enzymes. Colonies were selected on LB agar plates containing the appropriate antibiotics listed for each plasmid. Single colonies were used to inoculate 5-mL overnight starter cultures in LB with the appropriate antibiotic. The starter cultures were then used to inoculate 1 L of TB cultures containing the appropriate antibiotic, which were incubated at 37 °C with shaking until they reached the beginning of the exponential growth phase (an OD600 of approximately 0.4). Cultures were then cooled at 4 °C for 15 min prior to adding *L*-arabinose to a final concentration of 0.1%. Cultures were placed into a 20 °C incubator and shaken for at least 2 h until the culture reached the end of the exponential growth phase (an OD600 of approximately 1.2). The "slow growing time" for a minimum of 2 h before adding the next inducer was critical for the successful expression. Next, isopropyl β-D-1-thiogalactopyranoside (IPTG) was added to the culture to a final concentration of 0.5 mM. Cultures were placed into an 18 °C incubator and shaken overnight. Cells were harvested by centrifugation at 4,500 × g for 20 min. The culture media was decanted, and the cell pellet was stored at -80 °C prior to purification.

Epilancin 15X purification from *E. coli*
The cell pellet was suspended in 30 mL of lysis buffer (20 mM NaH_2PO_4, 500 mM NaCl, 0.5 mM imidazole, 20% glycerol, pH 7.5 at 25 °C) supplemented with Pierce Protease Inhibitor tablets (1 tablet per 1 L culture; Fisher Scientific) and benzonase (1 U for every 20 mL of lysate). The cell pellet was lysed by sonication (50% amplitude, 2 s pulse, 5 s pause, 15 min). Crude lysate was centrifuged at 24,000 ×g for 60 min at 4 °C and the supernatant was obtained. The supernatant was loaded onto a His60 Ni superflow resin (Takara) equilibrated with lysis buffer, and cell lysate was applied three times to it under the action of gravity. After loading the sample, the resin was washed with two column volumes (CV) of washing buffer (4 M guanidine hydrochloride, 20 mM NaH_2PO_4, 300 mM NaCl, 30 mM imidazole, pH 7.5 at 25 °C), and eluted using 1 CV of LanA Elution Buffer (4 M guanidine hydrochloride, 20 mM Tris HCl, pH 7.5 at 25 °C, 100 mM NaCl, 1

M imidazole). Peptides eluted from the Ni resin were concentrated and desalted using 3 kDa Amicon filters (Millipore). Lastly, the peptides were further purified with HPLC using an Agilent 1200 instrument (Agilent) as specified in general methods.

Leader peptide digestion by proteases

The cloning, expression, and purification of His_6-MBP-ElxP was performed as described previously,[3] resulting in 3 mg of purified ElxP protein per liter of cell culture. After incubating the modified His_6-ElxA with His_6-MBP-ElxP protease for 4 h at room temperature at pH 7.5, the digested peptide mass was confirmed by MALDI-TOF MS. The core peptides were further purified by HPLC using an Agilent 1200 instrument equipped with a Macherey-Nagel C18 reversed-phase column (4.6 mm i.d. × 250 mm L). A gradient of 0–70% solvent B (acetonitrile) in solvent A (20 mM ammonium acetate in water) over 30 min was used. The fractions corresponding to the epilancin 15X core peptides were confirmed with MALDI-TOF MS and lyophilized overnight.

Isolation of authentic epilancin 15X from *S. epidermidis* 15X154

Isolation of authentic epilancin 15X was performed as described previously with a few modifications.[3] In general, 1 L medium composed of Lab-Lemco meat extract (10%; Oxoid), malt extract (3%; Becton Dickinson), ammonium chloride (20 mM), $Ca(OH)_2$ (0.4%), NaCl (2%), 20 mL of 5 M HCl, final pH 6.5 was inoculated with an overnight preculture of *S. epidermidis* 15X154 in LB broth (1/100 dilution). A total of 1 L culture was incubated in two 4 L flasks (each flask contains 0.5 L culture) at 37 °C with shaking at 180 rpm for 15 h and harvested by centrifugation. Each 1 L supernatant was supplemented with one Pierce Protease Inhibitor tablet (Fisher Scientific) and stirred overnight at 4 °C. Solid $(NH_4)_2SO_4$ was added to the culture supernatant to reach 80% saturation (approximately 533 g per 1 L supernatant) at 4 °C and stirred overnight. The supernatant was centrifuged at 15,000 xg for 45 min, and the pellet was resuspended in a minimal amount of 50 mM $(NH_4)_2SO_4$ solution and filtered through a 0.45 μm filter (Millipore). The solution was loaded onto an HF Bond Elut C18 solid-phase extraction column (60 mL; Agilent). The column was washed with 20% acetonitrile in ammonium acetate buffer (20 mM, pH 5.5) to remove impurities, and the peptide was eluted with 95% acetonitrile in 0.1% trifluoroacetic acid (TFA). The eluted fractions were lyophilized overnight. The lanthipeptide was further purified using an ÄKTApurifier (Amersham Biosciences, GE Healthcare) equipped with a HisTrap™ SP HP cation exchange chromatography column (5 mL, Cytiva). After loading and washing the column with 10 CV of buffer A (50 mM HEPES, pH 7), a gradient of 0-100% buffer B (50 mM HEPES, 1M NaCl, pH 7) in buffer A over 10 CV was used to elute the peptide. The fractions containing epilancin 15X, as confirmed by MALDI-TOF MS, were combined and passed through the HF Bond Elut C18 solid-phase extraction column (10 mL; Agilent). The column was washed with 20% acetonitrile in water to remove impurities and the peptide was eluted with 95% acetonitrile in 0.1% (TFA). The eluted fractions were lyophilized overnight. The peptide was purified with HPLC using an Agilent 1200 instrument (Agilent) as specified in general methods. The fractions corresponding to the major peak were confirmed with MALDI-TOF MS and collected. A total of 10 mg of authentic epilancin 15X was obtained per liter of culture.

Antimicrobial activity assay of epilancin 15X

The autoclaved 1.5% agar was prepared in BHI media and cooled in a 40 °C water bath for 15 min. A total of 50 µL overnight culture of *S. carnosus* TM300 was added to the agar and kept in the water bath. A total of 25 mL seeded agar was poured into a sterile OmniTray (Nunc) and allowed to solidify at room temperature for 30 min. An additional 35 mL of LB seeded agar was poured over the lower solidified agar layer. A sterile 96-well PCR plate was placed in the molten agar upper layer, and the agar was allowed to solidify at 25 °C for 30 min. After sufficient solidification, the 96-well PCR plate was removed, and thus wells were made in the agar. Then 20 µL of each peptide was dispensed into wells. Plates were left at 25 °C for 30 min until the wells were dried, and the plates were incubated at 37 °C overnight. The antibacterial activity was determined by the presence or absence of growth inhibition.

Plasmid construction for heterologous expression of *Runella* and *Chryseobacterium* peptides

The lantibiotic biosynthetic gene clusters were refactored in pET28 plasmids as reported previously.[4] Correct assembly was first verified by restriction enzyme digestion and confirmed by Sanger sequencing. The $tRNA^{Glu}$ sequences were identified using the algorithm tRNAscan-SE,[1, 2] and the *Chryseobacterium* pEVOL plasmid was constructed by Twist.

Runella* and *Chryseobacterium* peptide purification from *E. coli

Cell pellet was suspended in 30 mL of lysis buffer (20 mM NaH_2PO_4, 500 mM NaCl, 0.5 mM imidazole, 20% glycerol, pH 7.5 at 25 °C) supplemented with lysozyme (100 µg/mL). The cell pellet was lysed by sonication (50% amplitude, 2 s pulse, 5 s pause, 15 min). Crude lysate was centrifuged at 24,000 ×g for 60 min at 4 °C and the supernatant was obtained. The supernatant was loaded onto a His60 Ni superflow resin (Takara) equilibrated with lysis buffer. After loading the filtered sample, the resin was washed with two column volumes (CV) each of washing buffer 1 (4 M guanidine hydrochloride, 20 mM NaH_2PO_4, 300 mM NaCl, 30 mM imidazole, pH 7.5) and washing buffer 2 (20 mM NaH_2PO_4, 300 mM NaCl, 30 mM imidazole, pH 7.5). The desired modified peptide(s) were eluted using 1–3 CV of Elution Buffer (20 mM NaH_2PO_4, pH 7.5, 100 mM NaCl, 1 M imidazole). Peptides eluted from the Ni resin were concentrated and desalted using 3 kDa Amicon filters (Millipore). A small sample of the eluted peptide was applied to a C18 ziptip and analyzed by MALDI-TOF-MS to check for peptide modifications.

Deglutathionylation using LanCL enzymes

The *in vitro* reaction contained 20 µM peptide and 20 µM LanCL protein (LanCL1 or LanCL2) in 25 mM Tris at pH 7.0 in H_2O. After the reaction was incubated at 25 °C for 30 min, 500 µM NEM was added to the reaction mixture which was further incubated for up to 24 h at 25 °C. An aliquot was desalted using a ziptip (C18) and the sample was analyzed by MALDI-TOF MS.

Non-enzymatic GSH addition to synthetic peptide RVS(Dha)YAV

A solution containing 100 µM of the synthetic 7-mer peptide RVS(Dha)YAV (reported previously)[5] was incubated with 1 mM GSH in 25 mM Tris at pH 9.0 in H_2O. After the reaction was incubated at 25 °C for 2 h, the mixture was loaded onto a HyperSep™ C18 cartridge (2000 mg, 15 mL, Thermo Scientific) to remove excess salt and GSH. The

glutathionylated peptide was eluted from the cartridge with 60% MeCN in H_2O. The resulting solution was lyophilized and analyzed by MALDI-TOF and LC/MS spectrometry. After desalting using a zip-tip (C18), samples were analyzed by MALDI-TOF mass spectrometry on a Bruker UltrafleXtreme spectrometer using a matrix solution containing 50 mg mL^{-1} Super-DHB (SDHB) in 3:2 MeCN/H_2O with 0.1% TFA.

LC/MS analysis of RVS(Dha)YAV, RVS(GS-A)YAV, and GS-NEM

For LC/MS analysis, the reaction mixture was filtered through a 0.2 µm Nanosep (Pall) and analyzed using a Shimadzu LC-20 HPLC and Shimadzu LCMS-2020. RVS(Dha)YAV, RVS(GS-A)YAV, and GS-NEM in the reaction mixture were analyzed using selected ion monitoring (SIM) at 763.4, 1070.4, and 433.1 Da, respectively. HPLC mobile phases were 0.1% TFA in water (HPLC solvent A) and 0.1% TFA in acetonitrile (HPLC solvent B). The sample was applied to a Xbridge C18 column (5 µm, 4.6 mm x 250 mm) equilibrated with 95% HPLC solvent A and 5% HPLC solvent B. A linear gradient to 95% HPLC solvent B was run over 20 min at 0.8 mL min^{-1}. The target peptides were eluted between 35–45% B.

Plasmid construction for heterologous expression of *P. citrea*.

The lantibiotic biosynthetic gene cluster was refactored with pET15 and pRSF plasmids using Gibson assembly (Figure S1). Correct assembly was confirmed by Sanger sequencing. The $tRNA^{Glu}$ sequence of *P. citrea* was identified using the algorithm tRNAscan-SE,[1, 2] and was not included in the expression system because of the high similarity to that of *E. coli* (Figure S4). Expressions were performed using BL21 star (DE3) cells to minimize the presence of glutathione adduct.

P. citrea* peptide purification from *E. coli

Cell pellet was suspended in 30 mL of lysis buffer (20 mM NaH_2PO_4, 6 M guanidine hydrochloride, 500 mM NaCl, 0.5 mM imidazole, pH 7.5 at 25 °C). The cell pellet was lysed by sonication (60% amplitude, 2 s pulse, 6 s pause, 5 min). Crude lysate was centrifuged at 50,000 ×g for 60 min at 4 °C and the supernatant was obtained. The supernatant was filtered and loaded onto a His60 Ni superflow resin (Takara) equilibrated with lysis buffer. After loading the filtered sample, the resin was washed with two column volumes (CV) each of lysis buffer, washing buffer 1 (4 M guanidine hydrochloride, 20 mM NaH_2PO_4, 500 mM NaCl, 30 mM imidazole, pH 7.5) and washing buffer 2 (20 mM NaH_2PO_4, 500 mM NaCl, 30 mM imidazole, pH 7.5). The desired modified peptides were eluted using 1–3 CV of Elution Buffer (20 mM NaH_2PO_4, pH 7.5, 100 mM NaCl, 1 M imidazole). Peptides eluted from the Ni resin were concentrated and desalted using 3 kDa Amicon filters (Millipore). A small sample of the eluted peptide was applied to a C18 ziptip and analyzed by MALDI-TOF-MS to check for peptide modifications.

LC/MS analysis of *P. citrea* peptide products

For LC/MS analysis, the modified PciA peptide was digested with AspN. The reaction mixture was applied to a TopTip C18 ziptip and the resulting sample was analyzed by LC/MS using an Agilent Infinity 1260 Liquid Chromatography instrument coupled to an

Agilent 6454 QTOF mass spectrometer using a Kinetex® 2.6 µm C8 100 Å, LC column (150 x 2.1 mm). HPLC mobile phases were 0.1% formic acid in water (HPLC solvent A) and 0.1% formic acid in acetonitrile (HPLC solvent B). The sample was applied to the column equilibrated with solvent A. The peptides were eluted using the following gradient: 0-5% B over 3 min, 5-50% B over 10 min, and 50-95% B over 1 min for a total run time of 14 min.

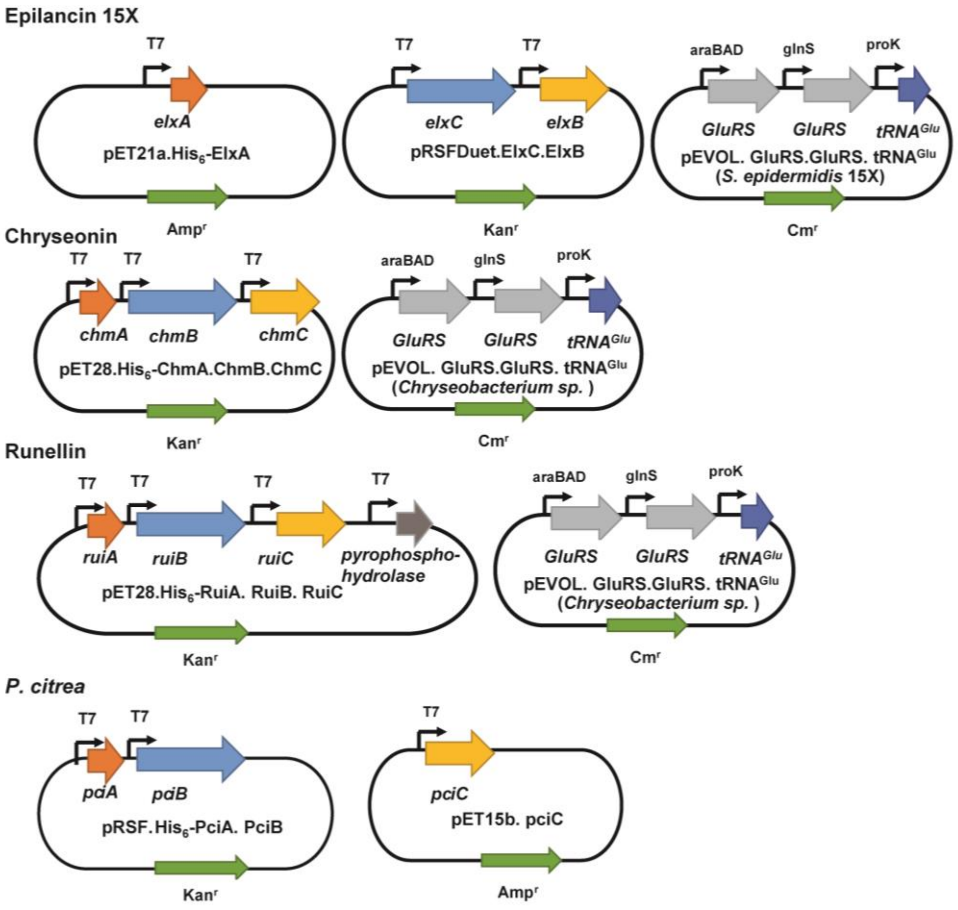

Figure S1. Schematic representation of the production systems for epilancin 15X, and the peptides from *Chryseobacterium*, *Runella*, and *P. citrea* used in this study. In order to generate the mature peptides from the latter three products, peptidase digestion is needed to remove the leader peptide. Unfortunately, use of the substrate tolerant protease LahT150[6] was unsuccessful with these modified peptides.

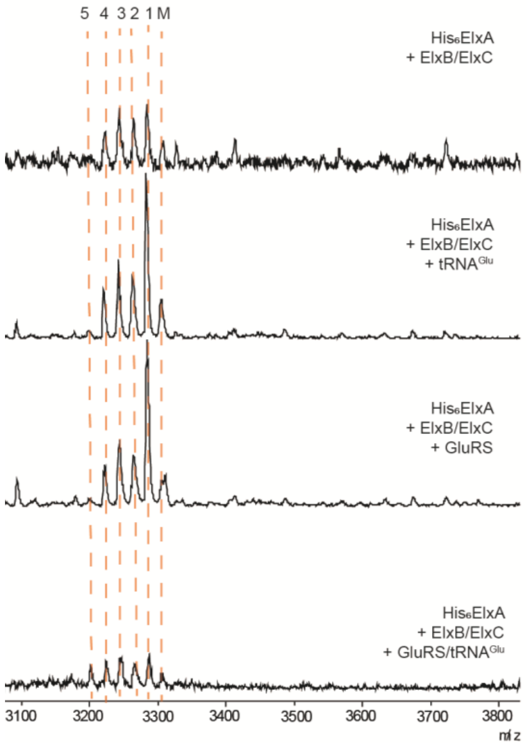

Figure S2. MALDI-TOF MS analysis of heterologous expression of ElxA with the components shown in the various panels and after *in vitro* leader peptide removal by ElxP. All peptides/proteins were encoded under T7 promoters without using pEVOL plasmids. No fully eight-fold dehydrated product was observed. M, unmodified ElxA ($[M+H]^+$ *m/z* obs. 3318, calc. *m/z* 3317); 1, 1-fold dehydrated ElxA ($[M+H]^+$ *m/z* obs. 3299, calc. *m/z* 3299); 2, 2-fold dehydrated ElxA ($[M+H]^+$ *m/z* obs. 3281, calc. *m/z* 3282); 3, 3-fold dehydrated ElxA ($[M+H]^+$ *m/z* obs. 3263, calc. *m/z* 3263); 4, 4-fold dehydrated ElxA ($[M+H]^+$ *m/z* obs. 3246, calc. *m/z* 3245); 5, 5-fold dehydrated ElxA ($[M+H]^+$ *m/z* obs. 3227, calc. *m/z* 3228)).

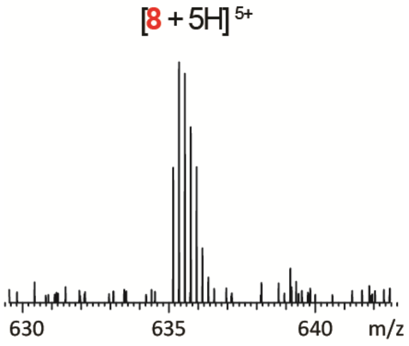

Figure S3. Electrospray ionization MS analysis of RP-HPLC-purified fully dehydrated ElxA after *in vitro* leader peptide removal by ElxP. 8, 8-fold dehydrated ElxA ($[M+5H]^{5+}$ *m/z* obs. 635.1439, calc. *m/z* 635.1484, ppm error −7.08).

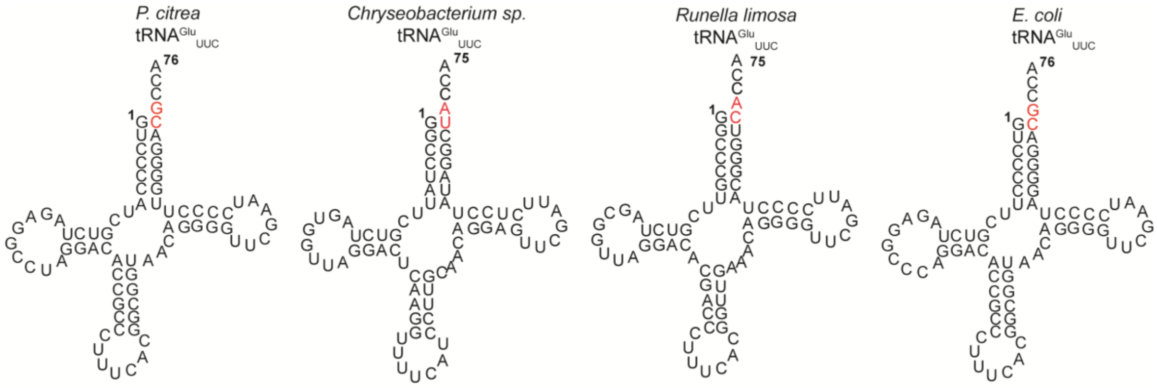

Figure S4. Predicted cloverleaf structures of *Pseudoalteromonas citrea*, *Chryseobacterium* sp., *Runella limosa*, and *E. coli* tRNA^{Glu} made using the tRNAscan-SE algorithm.[7, 8] The previously identified recognition elements of the lanthipeptide dehydratases on the tRNA^{Glu} acceptor stem are highlighted in red.

Chryseobacterium sp. OV715

GluC-digested peptide:

VGVSKDFDTPTFNDKCFDGGSTTFRKACTWCGFFHSTKW-7H_2O+2GSH

Formula: C220 H304 N57 O63 S5

$[M+H]^+$ = 4912.0929

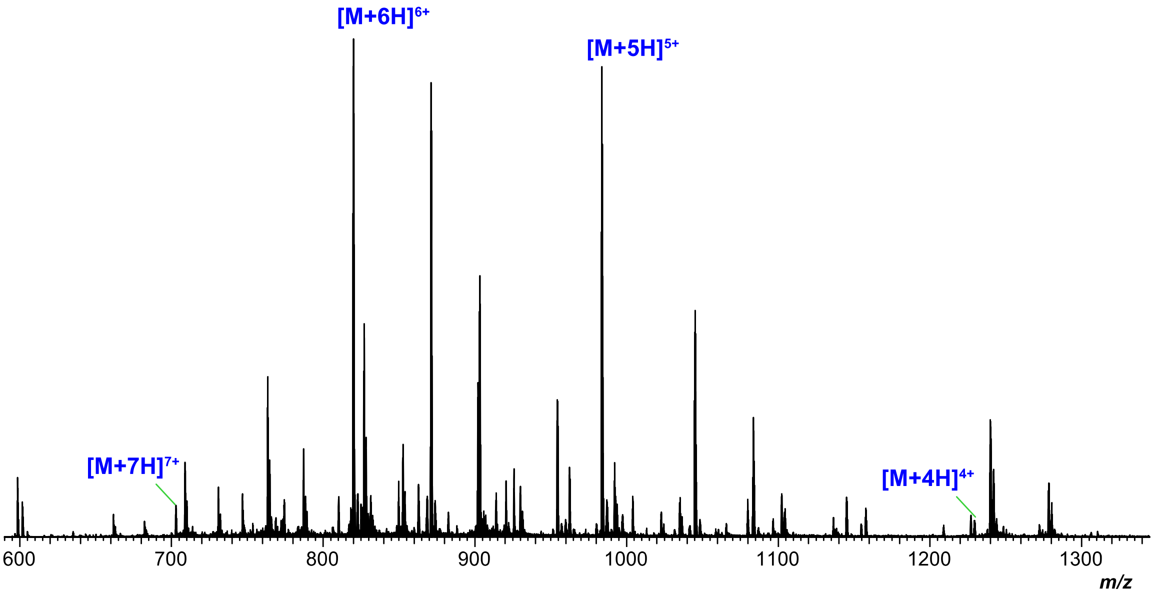

ion	Calc. Mass	Obs. Mass	Error (ppm)
$[M+4H]^{4+}$	1228.7787	1228.7854	5.5
$[M+5H]^{5+}$	983.2244	983.2280	3.7
$[M+6H]^{6+}$	819.5215	819.5243	3.4
$[M+7H]^{7+}$	702.5909	702.5927	2.6

Figure S5. HRMS of modified ChmA digested with endoproteinase GluC. ESI-qTOF MS (Orbitrap) spectrum of GluC-digested seven-fold dehydrated ChmA carrying two GSH adducts. Observed and calculated masses are shown in the table.

***Chryseobacterium* sp. OV715**

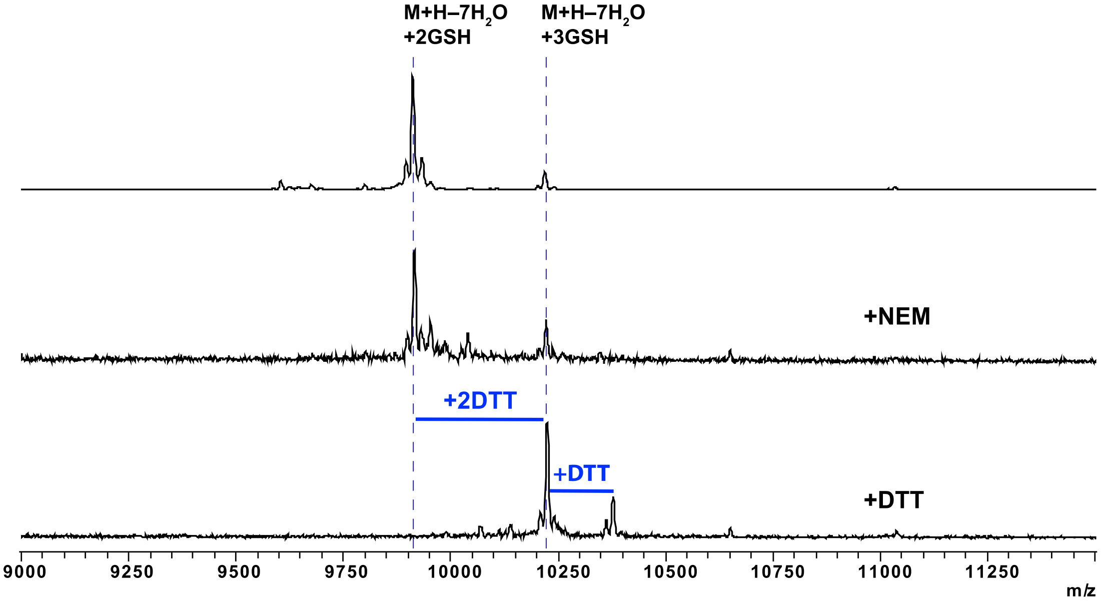

Figure S6. NEM alkylation and DTT addition assays of the glutathionylated mChmA.

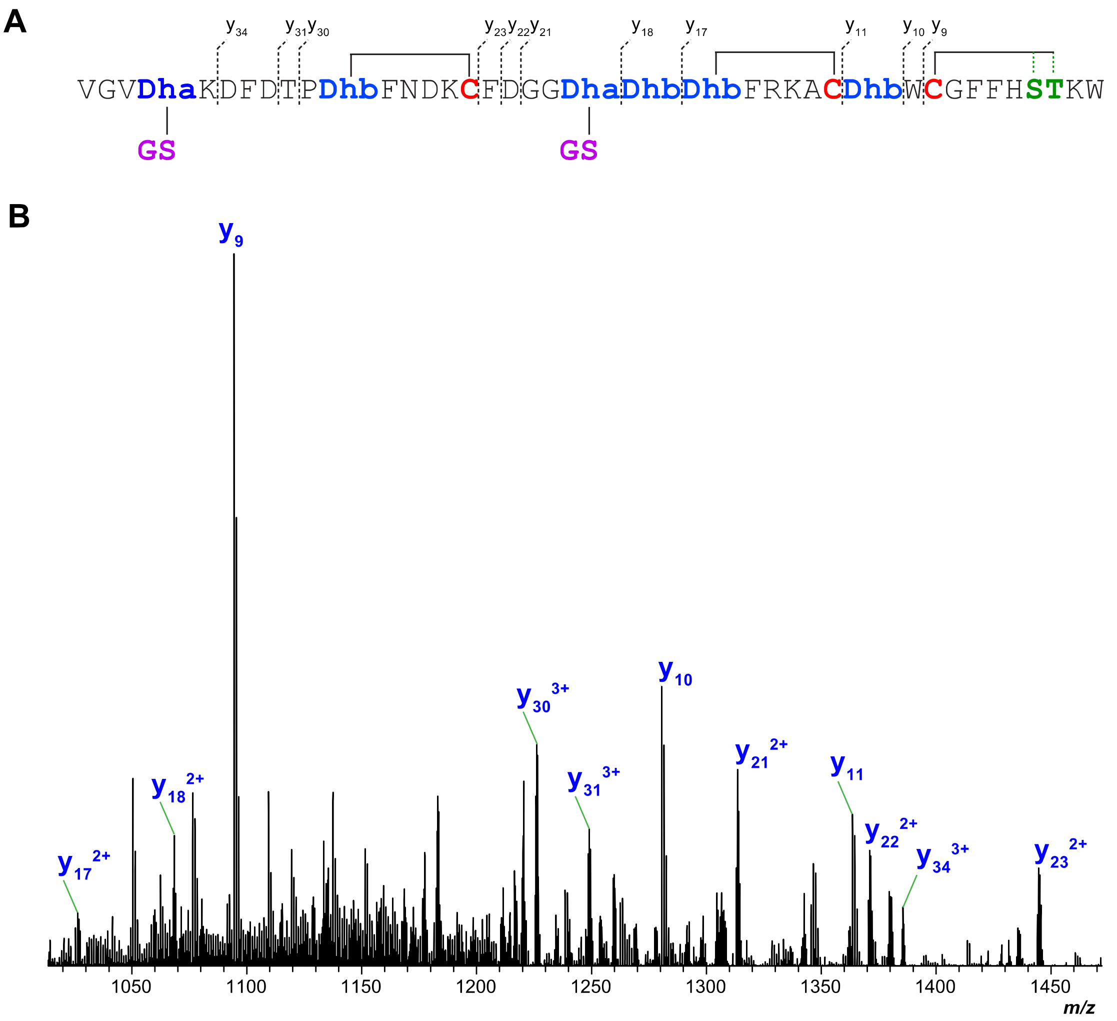

C

ion	Calc. Mass	Obs. Mass	Error (ppm)
$y9^{+}$	1094.4876	1094.4885	0.8
$y10^{+}$	1280.5670	1280.5674	0.3
$y11^{+}$	1363.6041	1363.6044	0.2
$y17^{2+}$	1026.4796	1026.4785	1.1
$y18^{2+}$	1067.9982	1068.0023	3.8
$y21^{2+}$	1313.0721	1313.0730	0.7

ion	Calc. Mass	Obs. Mass	Error (ppm)
$y22^{2+}$	1370.5856	1370.5858	0.1
$y23^{2+}$	1444.1198	1444.1202	0.3
$y30^{3+}$	1225.5264	1225.5282	1.5
$y31^{3+}$	1259.2090	1259.2054	2.9
$y34^{3+}$	1384.9164	1384.9206	3.0

Figure S7. HR-ESI tandem mass spectrometry analysis of modified ChmA digested with endoproteinase GluC. (A) Core peptide sequence and observed MS/MS ions. Green dotted lines indicate possible covalent bonds of Cys to either a dehydrated Ser or Thr which cannot be distinguished with the current data. (B) Tandem mass spectrum; (C) Observed and calculated masses for fragments. GS, glutathione adduct.

Runella limosa

His-tagged full-length peptide:

MHHHHHHAMKKQLSKLNLKTDKVVNLSKDNSQGVVGGRPQSNPNSKVYCLSDLCTFTGATFSC−$7H_2O$+3GSH

Formula: C329 H525 N101 O102 S8

$[M+H]^+$ = 7768.6215

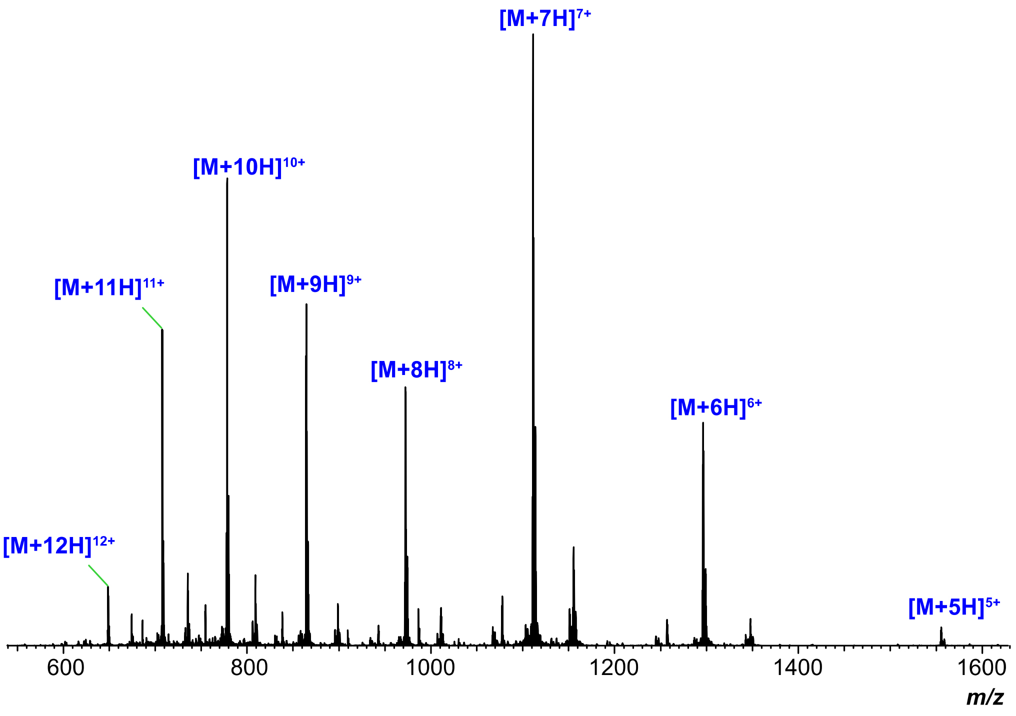

ion	Calc. Mass	Obs. Mass	Error (ppm)
$[M+5H]^{5+}$	1554.5301	1554.5326	1.6
$[M+6H]^{6+}$	1295.6096	1295.6128	2.5
$[M+7H]^{7+}$	1110.6664	1110.6698	3.1
$[M+8H]^{8+}$	971.9591	971.9612	2.4
$[M+9H]^{9+}$	864.0755	864.0785	3.5
$[M+10H]^{10+}$	777.7687	777.7718	4.0
$[M+11H]^{11+}$	707.1540	707.1560	2.8
$[M+12H]^{12+}$	648.3085	648.3091	0.9

Figure S8. HRMS of modified RuiA. ESI-qTOF MS (Orbitrap) spectrum of modified RuiA (M – 7 H_2O + 3 GSH) (indicated in the spectra as M). Observed and calculated masses for the peptide are shown in the table.

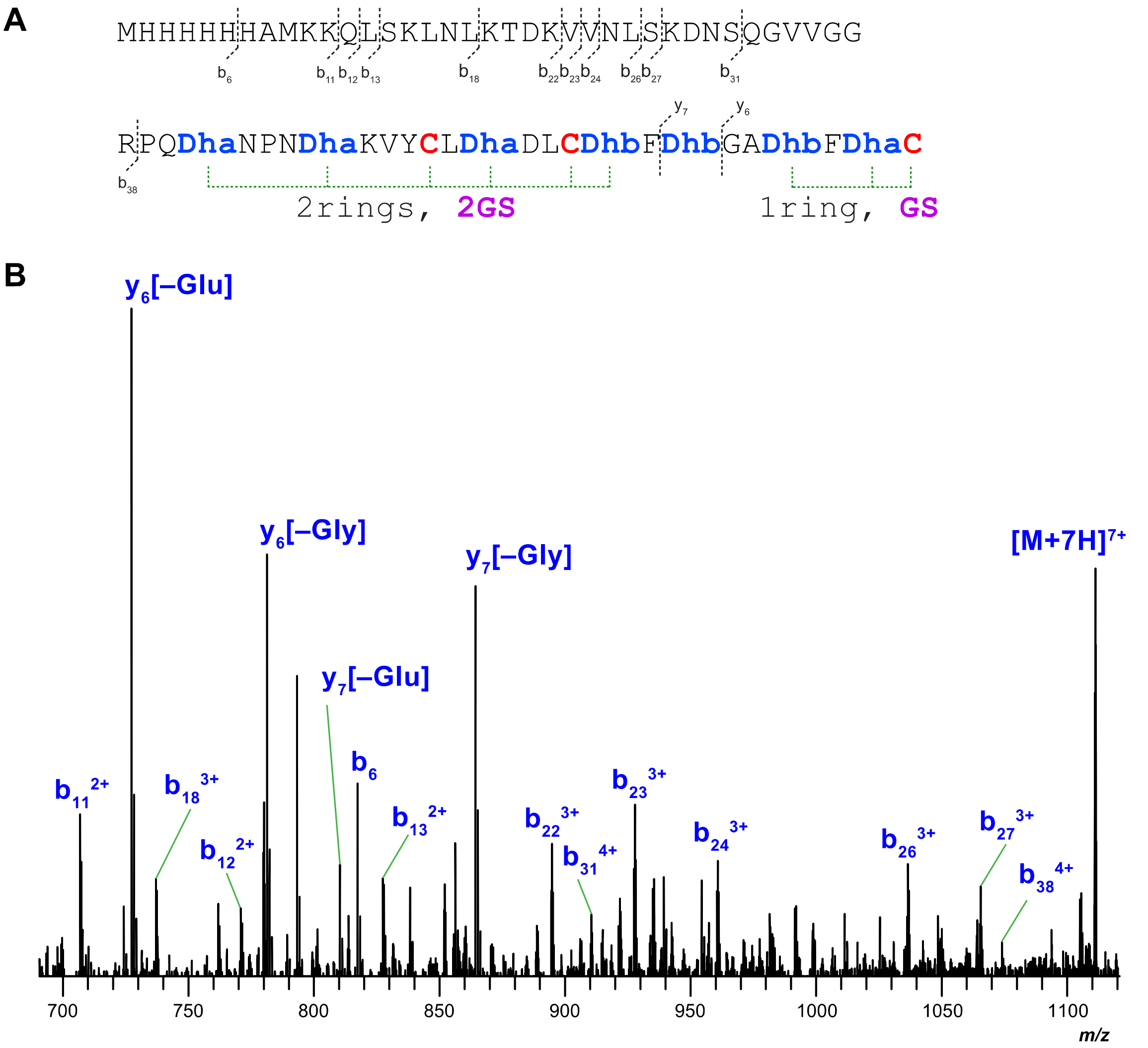

C

ion	Calc. Mass	Obs. Mass	Error (ppm)
b6^{+}	817.3423	817.3435	1.5
b11^{2+}	706.8380	706.8382	0.3
b12^{2+}	770.8673	770.8675	0.3
b13^{2+}	827.4093	827.4088	0.6
b18^{3+}	737.0547	737.0553	0.8
b22^{3+}	894.4762	894.4790	3.1
b23^{3+}	927.4990	927.4986	0.4
b24^{3+}	960.5218	960.5233	1.6

ion	Calc. Mass	Obs. Mass	Error (ppm)
b26^{3+}	1036.2308	1036.2373	6.3
b27^{3+}	1065.2415	1065.2443	2.6
b31^{4+}	910.2321	910.2331	1.1
b38^{4+}	1384.9164	1384.9206	3.0
y6[−Glu]$^{+}$	727.2538	727.2545	1.0
y6[−Gly]$^{+}$	781.2644	781.2651	0.9
y7[−Glu]$^{+}$	810.2909	810.2920	1.4
y7[−Gly]$^{+}$	864.3015	864.3029	1.6

Figure S9. HR-ESI tandem mass spectrometry analysis of modified RuiA (mRuiA) (A) Peptide sequence and observed MS/MS ions. Green dotted lines indicate possible covalent bonds. Between the b_{38} and y_7 fragments, two dehydroamino acids are involved in (methyl)lanthionine ring formation with two Cys and the other two dehydro amino acids are glutathionylated; (B) Tandem mass spectrum. [−Glu] or [−Gly] means that the Glu or Gly residue of glutathione is removed from the corresponding fragment during tandem MS; (C) Observed and calculated masses for fragments.

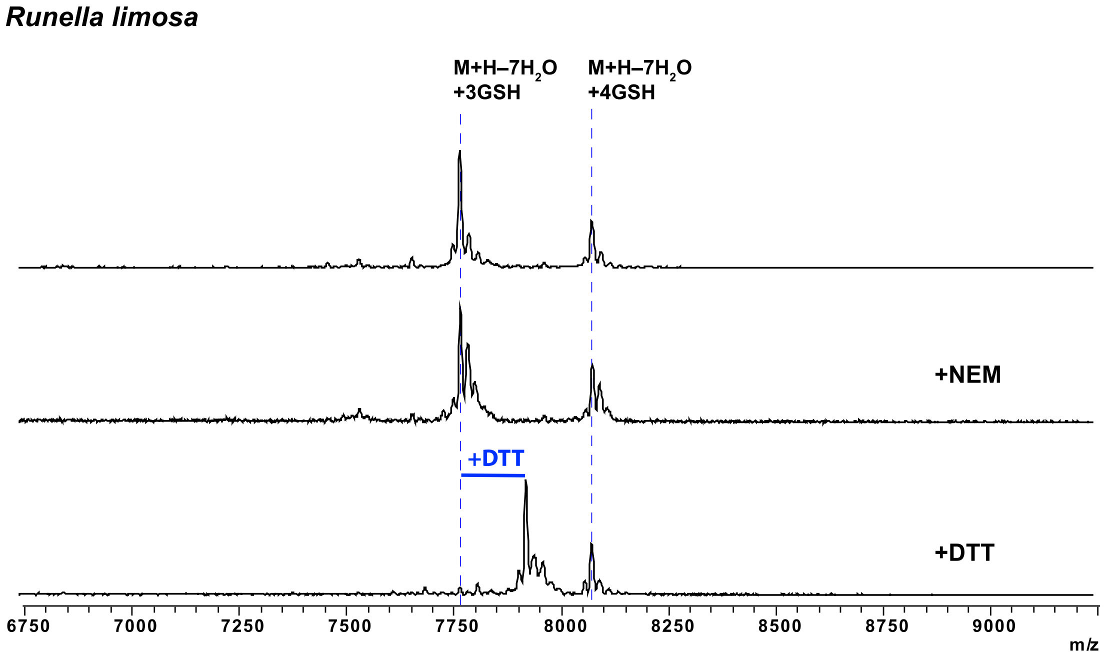

Figure S10. NEM and DTT alkylation assays of the glutathionylated mRuiA.

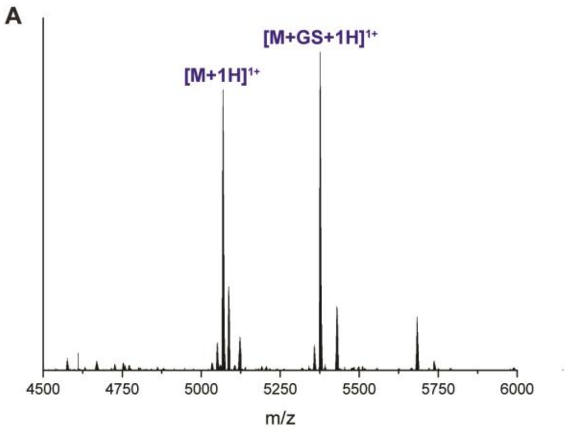

Ion	Calc. Mass	Obs. Mass	Error (ppm)
$[M+1H]^{1+}$	5067.2634	5067.2867	4.6
$[M+GS+1H]^{1+}$	5374.3472	5374.3751	5.2

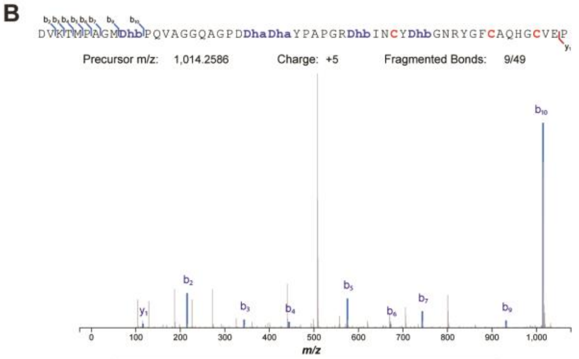

Ion	Calc. Mass	Obs. Mass	Error (ppm)
y_1^+	116.0706	116.0708	1.7
b_2^+	215.1026	215.103	1.7
b_3^+	343.1976	343.1974	-0.6
b_4^+	444.2453	444.2477	5.5
b_5^+	575.2858	575.2864	1.1
b_6^+	672.3385	672.3406	3.1
b_7^+	743.3756	743.3767	1.4
b_9^+	931.4376	931.4372	-0.4
b_{10}^+	1014.4747	1014.4706	-4.0

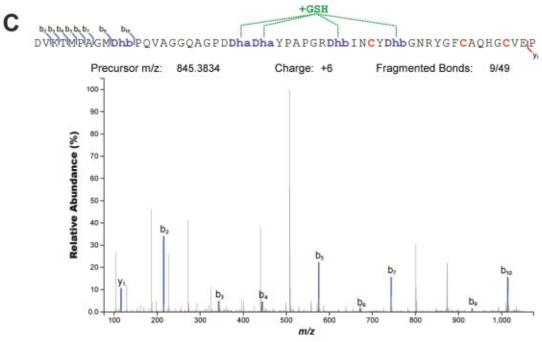

Ion	Calc. Mass	Obs. Mass	Error (ppm)
y_1^+	116.0706	116.0704	-1.8
b_2^+	215.1026	215.1022	-2.0
b_3^+	343.1976	343.1964	-3.5
b_4^+	444.2453	444.2453	0.1
b_5^+	575.2858	575.2856	-0.3
b_6^+	672.3385	672.3401	2.4
b_7^+	743.3756	743.3757	0.1
b_9^+	931.4376	931.43	-8.1
b_{10}^+	1014.4747	1014.4736	-1.1

Figure S11. High resolution mass spectrum of posttranslationally modified PciA. (A) Deconvoluted MS spectrum taken from peptide isolated from heterologous expression after digestion with AspN. (B) Tandem MS spectrum showing fragmentation of the +5 ion of AspN-digested PciA not containing glutathione adducts (precursor m/z = 1014.42586 Da). (C) Tandem MS spectrum showing fragmentation of the +6 ion of AspN-digested PciA containing one glutathione adduct (precursor m/z = 845.3834 Da). Locations of three (methyl) lanthionine rings (not shown) and glutathione adduct (green) could not be definitively assigned because of lack of internal fragmentation.

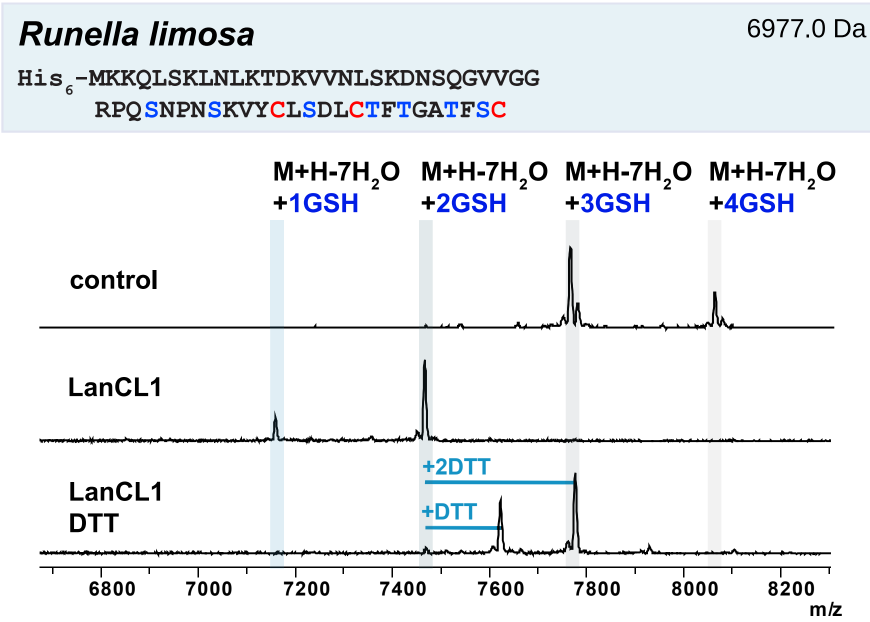

Figure S12. MALDI-TOF mass spectrum of glutathionylated mRuiA. DTT alkylation assay of the peptide was performed after the treatment with LanCL1.

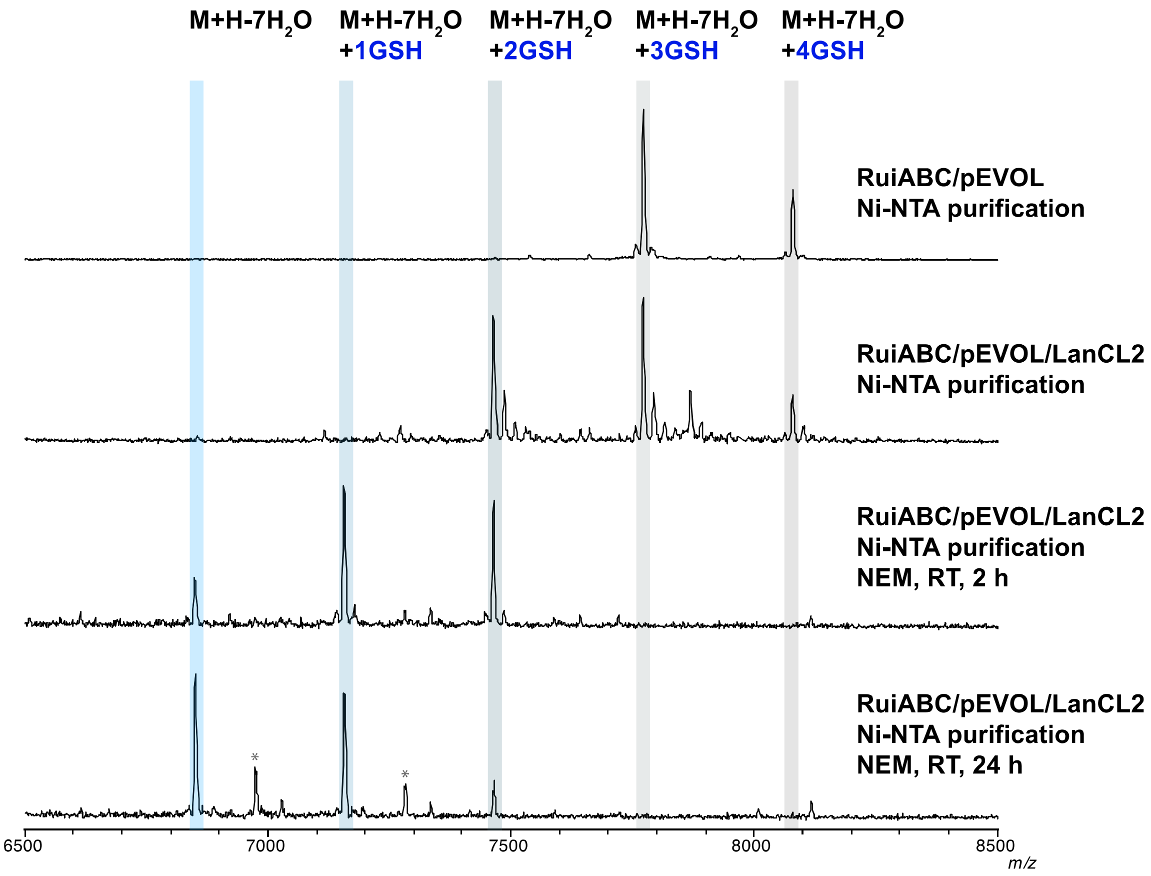

Figure S13. MALDI-TOF MS data from co-expression of the refactored gene cluster from *Runella limosa* in pET28(RuiABC), tRNA^{Glu} and GluRS from *Chryseobacterium* sp. OV715 in pEVOL(tRNA^{Glu}/GluRS), and human LanCL2 in pET28(LanCL2). After Ni-NTA purification, the buffer was exchanged to 20 mM Tris (pH 7.5) for the data shown in the third and fourth panels. Then 200 μM NEM was added to the sample which was incubated for 2 h or 24 h. Asterisks in the last panel indicate NEM adducts of mRuiA.

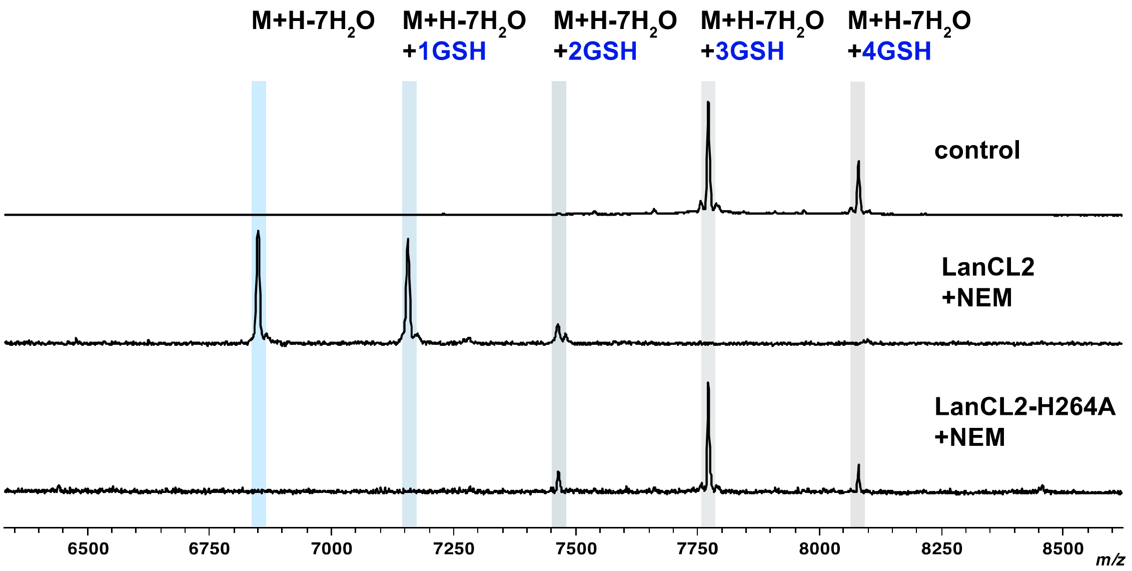

Figure S14. MALDI-TOF mass spectra of the use of wt LanCL2 or LanCL2-H264A for deglutathionylation of modified RuiA in the presence of NEM.

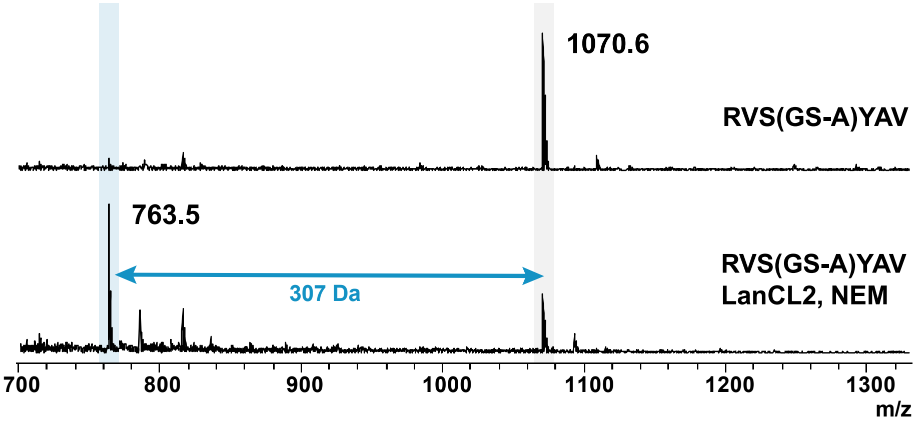

Figure S15. MALDI-TOF mass spectrum of glutathionylated and SPE-purified synthetic heptamer RVS(GS-A)YAV after the treatment of LanCL2 and NEM. GS-A, glutathione adduct to former Dha.

Table S1. MALDI-TOF MS data from heterologous expression of pET21a(ElxA) and pRSFDuet(ElxBC) from *S. epidermidis* 15X154. (–H_2O: the number of dehydrations; Calc. Mass: calculated mass $[M+H]^+$; Obs. Mass: observed mass $[M+H]^+$).

–H_2O	Calc. Mass	Obs. Mass
8	3173	3172
7	3191	3191
6	3209	3208
5	3227	3227
4	3245	3246
3	3263	3263
2	3281	3281
1	3299	3299
0	3317	3318

Table S2. MALDI-TOF MS data from heterologous expression of pET28(ChmABC) from *Chryseobacterium* sp. OV715. (+GSH: the number of glutathionylations; $–H_2O$: the number of dehydrations; Calc. Mass: calculated mass $[M+H]^+$; Obs. Mass: observed mass $[M+H]^+$)

+GSH	$–H_2O$	Calc. Mass	Obs. Mass
0	5	9334.8	9335.1
	4	9352.8	9352.3
	3	9370.8	9369.4
	2	9388.8	9387.9
	1	9406.8	9404.5
1	7	9606.1	9604.1
	6	9624.1	9624.0
	5	9642.1	9642.0
	4	9660.1	9659.1
	3	9678.1	9676.6
	2	9696.1	9693.2
2	7	9913.4	9910.9
	6	9931.4	9931.6
	5	9949.4	9949.2
	4	9967.4	9965.1
	3	9985.4	9982.5
	2	10003.5	10002.5
3	7	10220.0	10218.7
	6	10238.0	10239.1
	5	10256.0	10255.2
	4	10274.0	10271.3
	3	10292.1	10292.0

Table S3. MALDI-TOF MS data from heterologous expression of pET28(RuiABC) from *Runella limosa* in *E. coli*. (+GSH: the number of glutathionylation; $-H_2O$: the number of dehydrations; Calc. Mass: calculated mass $[M+H]^+$; Obs. Mass: observed mass $[M+H]^+$)

+GSH	$-H_2O$	Calc. Mass	Obs. Mass
0	4	6906.0	6907.1
	3	6924.0	6925.0
	2	6942.0	6942.6
	1	6960.0	6959.1
1	6	7177.3	7179.6
	5	7195.3	7196.2
	4	7213.3	7214.2
	3	7231.3	7231.1
	2	7249.3	7248.5
2	7	7466.1	7468.8
	6	7484.1	7485.9
	5	7502.1	7503.8
	4	7520.1	7521.3
3	7	7773.2	7776.3
	6	7791.2	7792.7
	5	7809.2	7810.1
4	7	8080.3	8081.7

Table S4. Strains and plasmids used in this study.

Strains or plasmids	Purpose	Source or reference
Strains		
E. coli BL21(DE)	Heterologous expression strain	NEB
E. coli DH5α	Plasmid propagation strain	NEB
E. coli Rosetta2(DE3)	Heterologous expression strain	Novagen
Staphylococcus epidermidis 15X154	Epilancin 15X producer strain	Ref[9]
Staphylococcus carnosus TM300	Epilancin 15X sensitive strain	G. Bierbaum, U. of Bonn
Plasmids		
pET21a_His$_6$ElxA	Overexpression of ElxA in *E. coli*	This study
pRSFDuet_ElxC_ElxB	Overexpression of ElxB and ElxC in *E. coli*	This study
pET28a_His$_6$ChmA_ChmB_ChmC	Overexpression of ChmA, ChmB, and ChmC in *E. coli*	This study
pET28a_His$_6$RuiA_RuiB_RuiC	Overexpression of RuiA, RuiB, and RuiC in *E. coli*	This study
pEVOL_GluRS_GluRS_tRNA^{Glu} (*S. epidermidis* 15X154)	Overexpression of *S. epidermidis* 15X154 GluRS and tRNA^{Glu} under various promoters in *E. coli*	This study
pEVOL_ GluRS_GluRS_tRNA^{Glu} (*Chryseobacterium* sp. OV715)	Overexpression of *Chryseobacterium* sp. OV715 GluRS and tRNA^{Glu} under various promoters in *E. coli*	This study
pEVOL.pAzF	pEVOL empty plasmid as the negative control	Addgene

Table S5. Primers used in this study.

Name	5' Sequence 3'
**Epilancin 15X**	
pET21a_elxA_gBlock_F	GGATCGAGATCTTAATACGACTCACTATAGGG
pET21a_elxA_gBlock_R	CTCGAGTGCGGCCGCTTATTTTTACCAGTAAAGTGAC
pET21a_elxA_BB_F	GGTAAAAAATAAGCGGCCGC
pET21a_elxA_BB_R	GATCTCGATCCTCTACGCC
pEVOL_GlutRNA (Sepidermidis15X)_BB_R	GTCAATTATTACCTCCACGGGG
pEVOL_GlutRNA (Sepidermidis15X)_BB_F	CGAATTTGCTTTCGAATTTCTGCC
pEVOL_GlutRNA (Sepidermidis15X)_gblock_F	GGAGGTAATAATTGACGATATGATCAGTGCACGGCTAACTAAGCGG

pEVOL_GlutRNA (Sepidermidis15X)_gblock_R	GCAAATTCGACCCTGAGCTGCTCGAGCATGCAAAAAAGCCTG
pEVOL_araBAD_BglII_gluRS (Sepidermidis15X)_F	CTAACAGGAGGAATTAGATCTATGAGTGAACGTATCAGAG
pEVOL_araBAD_gluRS (Sepidermidis15X)_SalI_R	CTCAATGATGATGATGATGATGGTCGACTTAAACAAGTTTTTTTAAGCGTG
pEVOL_glnS_NdeI_gluRS (Sepidermidis15X)_F	GTTGTTTACGCTTTGAGGAATCCCATATGAGTGAACGTATCAGAG
pEVOL_glnS_gluRS (Sepidermidis15X)_PstI_R	CAATTTAGCGTTTGAAACTGCAGTTAAACAAGTTTTTTTAAGCGTG
pRSF_NcoI_elxC_F	CTTTAATAAGGAGATATACCATGGGCATGGAAAATAGTATCCAAAAATCCTTATC
pRSF_elxC_NotI_R	GTATAAATTGTTTTGTTTTGCGTAAgcggccgcataatgcttaag
pRSF_BglII_elxB_F	GAAGGAGATATACATATGGCAGATCTCATGAACATCTTCAAAAAAT
pRSF_elxB_XhoI_R	CGGTTTCTTTACCAGACTCGAGTTAGTTGATTTTTTTGTAG
pEVOL_tRNAGlu (Sepidermidis15X)_gBlock	GTGCACGGCTAACTAAGCGGCCTGCTGACTTTCTCGCCGATCAAAAGGCATTTTGCTATTAAGGGATTGACGAGGGCGTATCTGCGCAGTAAGATGCGCCCCGCATTGGCTCCTTGGTCAAGCGGTtAAGACACCGCCCTTTCACGGCGGTAaCACGGGTTCGAGTCCCGTAGGAGTCACCAAATTCGAAAAGCCTGCTCAACGAGCAGGCTTTTTTGCATGCTCGAG
pET21a_elxA_gBlock	TAATACGACTCACTATAGGGGAATTGTGAGCGGATAACAATTCCCCCTGTAGAAATAATTTTGTTTAACTTTAATAAGGAGATATACCATGGGCAGCAGCCATCACCATCATCACCACAGCCAGGATCCGATGAAAAAAGAATTATTTGATTTAAATCTTAATAAAGATATCGAGGCACAAAAAAGTGACCTAAATCCGCAATCAGCTAGTATTGTTAAAACAACTATCAAAGCTTCTAAAAAGCTTTGTAGAGGATTTACACTAACTTGTGGATGTCACTTTACTGGTAAAAAATAA

References

1. T. M. Lowe and S. R. Eddy, *Nucleic Acids Res.*, 1997, **25**, 955-964.
2. P. P. Chan and T. M. Lowe, *Methods Mol. Biol.*, 2019, **1962**, 1-14.
3. J. E. Velásquez, X. Zhang and W. A. van der Donk, *Chem. Biol.*, 2011, **18**, 857-867.
4. R. S. Ayikpoe, C. Shi, A. J. Battiste, S. M. Eslami, S. Ramesh, M. A. Simon, I. R. Bothwell, H. Lee, A. J. Rice, H. Ren, Q. Tian, L. A. Harris, R. Sarksian, L. Zhu, A. M. Frerk, T. W. Precord, W. A. van der Donk, D. A. Mitchell and H. Zhao, *Nat. Commun.*, 2022, **13**, 6135.
5. M. N. Lundahl, R. Sarksian, H. Yang, R. J. Jodts, A. Pagnier, D. F. Smith, M. A. Mosquera, W. A. van der Donk, B. M. Hoffman, W. E. Broderick and J. B. Broderick, *J. Am. Chem. Soc.*, 2022, **144**, 5087-5098.
6. S. C. Bobeica, S. H. Dong, L. Huo, N. Mazo, M. I. McLaughlin, G. Jimenéz-Osés, S. K. Nair and W. A. van der Donk, *eLife*, 2019, **8**, e42305.
7. T. M. Lowe and P. P. Chan, *Nucleic Acids Res.*, 2016, **44**, W54-57.
8. P. P. Chan, B. Y. Lin, A. J. Mak and T. M. Lowe, *Nucleic Acids Res.*, 2021, **49**, 9077-9096.
9. M. B. Ekkelenkamp, M. Hanssen, S. T. Danny Hsu, A. de Jong, D. Milatovic, J. Verhoef and N. A. van Nuland, *FEBS Lett.*, 2005, **579**, 1917-1922.
